# EBV epitranscriptome reprogramming by METTL14 is critical for viral-associated tumorigenesis

**DOI:** 10.1371/journal.ppat.1007796

**Published:** 2019-06-21

**Authors:** Fengchao Lang, Rajnish Kumar Singh, Yonggang Pei, Shengwei Zhang, Kunfeng Sun, Erle S. Robertson

**Affiliations:** Departments of Otorhinolaryngology-Head and Neck Surgery, and Microbiology, the Tumor Virology Program, Abramson Cancer Center, Perelman School of Medicine at the University of Pennsylvania, Philadelphia, Pennsylvania, United States of America; Brigham and Women's Hospital, UNITED STATES

## Abstract

Epstein–Barr virus (EBV) is a ubiquitous oncogenic virus that induces many cancers. N6-Methyladenosine (m6A) modification regulates many cellular processes. We explored the role of m6A in EBV gene regulation and associated cancers. We have comprehensively defined m6A modification of EBV latent and lytic transcripts. Furthermore, m6A modification demonstrated a functional role in regulation of the stability of viral transcripts. The methyltransferase METTL14 was induced at the transcript and protein levels, and knock-down of METTL14 led to decreased expression of latent EBV transcripts. METTL14 was also significantly induced in EBV-positive tumors, promoted growth of EBV-transformed cells and tumors in Xenograft animal models. Mechanistically, the viral-encoded latent oncoprotein EBNA3C activated transcription of METTL14, and directly interacted with METTL14 to promote its stability. This demonstrated that EBV hijacks METTL14 to drive EBV-mediated tumorigenesis. METTL14 is now a new target for development of therapeutics for treatment of EBV-associated cancers.

## Author summary

Epstein–Barr virus (EBV) is a ubiquitous oncogenic virus that contributes to approximately 2% of all cancers by modulating a myriad of host cell activities. M6A is the most abundant RNA modification which is important for infection with HIV-1, HCV, Zika virus, KSHV and SV40 virus. The role of m6A modification and the associated enzymes in EBV infection and related oncogenic activities has not been explored. We investigated the role of m6A modification on EBV-infection and examined its effects on the proliferative activities of the virus which contributes to cancer. We showed that the m6A methyltransferase METTL14 is an important factor in EBV-induced oncogenesis. METTL14 was dramatically increased in EBV latently infected cells. Knock-down of METTL14 led to decreased latent viral gene expression. The essential EBV latent antigen EBNA3C was up-regulated by METTL14-mediated m6A modification, and its expression led to a feedback loop in up-regulation of METTL14 transcription, and the stability of its protein. We now provide a more comprehensive understanding of EBV and the host cell RNA processing machinery critical for regulation of EBV activities to maintain latent infection and its oncogenic properties.

## Introduction

Epstein-Barr virus (EBV or HHV4), was the first human oncogenic virus discovered and isolated from a Burkitt’s lymphoma patient in 1964 [1]. EBV contributes to approximately 2% of all cancers [2]. EBV exhibits two distinct phases, lytic infection and latent infection. Latent infection is referred to as latency 0, I, II and III [3]. Viral protein expression during latency III can potently promote indefinite proliferation of primary B-cells ultimately mediating immortalization of infected cells [4].

N6 methylation of adenosine (m6A) is the most abundant RNA modification and it is distributed at translation start sites, CDS regions, and 3’ UTRs [5–7]. Deposition of m6A on RNA is controlled by the cellular m6A machinery, which is the methyltransferase complex (METTL3, METTL14 and WTAP) and the demethylase enzymes (FTO and ALKBH5) [8]. Studies have shown that m6A modification has important roles during infection of cells with HIV-1 [9], HCV [10], Zika virus [11], KSHV [12] and SV40 virus [13]. However, the role of m6A modification and the associated enzymes on infection by the ubiquitous human tumor virus EBV, and the effects on its potent oncogenic activities have not been explored.

We investigated the role of m6A modification on EBV-mediated cell transformation, and further examined its contribution to the tumorigenic activities of the virus. We demonstrated that m6A modification of the EBV epitranscriptome facilitated enhanced expression of its latent genes and repressed lytic gene expression. Furthermore, we showed that the m6A methyltransferase METTL14 is a critical factor required for EBV-induced oncogenesis. We also showed that METTL14 was dramatically increased in EBV latently infected cells and down-regulated during EBV lytic infection. Knock-down of METTL14 led to decreased latent gene expression and an increase in lytic gene expression. EBV latent antigen EBNA3C, which is critical for viral-mediated transformation of cells, was up-regulated by METTL14-mediated m6A modification, and its expression led to a feedback loop by which METTL14 transcription was also induced, as well as its protein stability. We now provide for the first time a comprehensive understanding of the EBV epitranscriptome, and the host cell RNA processing machinery critical for regulation of viral transcripts to maintain latent infection required for its oncogenic properties.

## Results

### EBV latent transcripts were predominantly marked with m6A during latent infection

**Fig 1:**
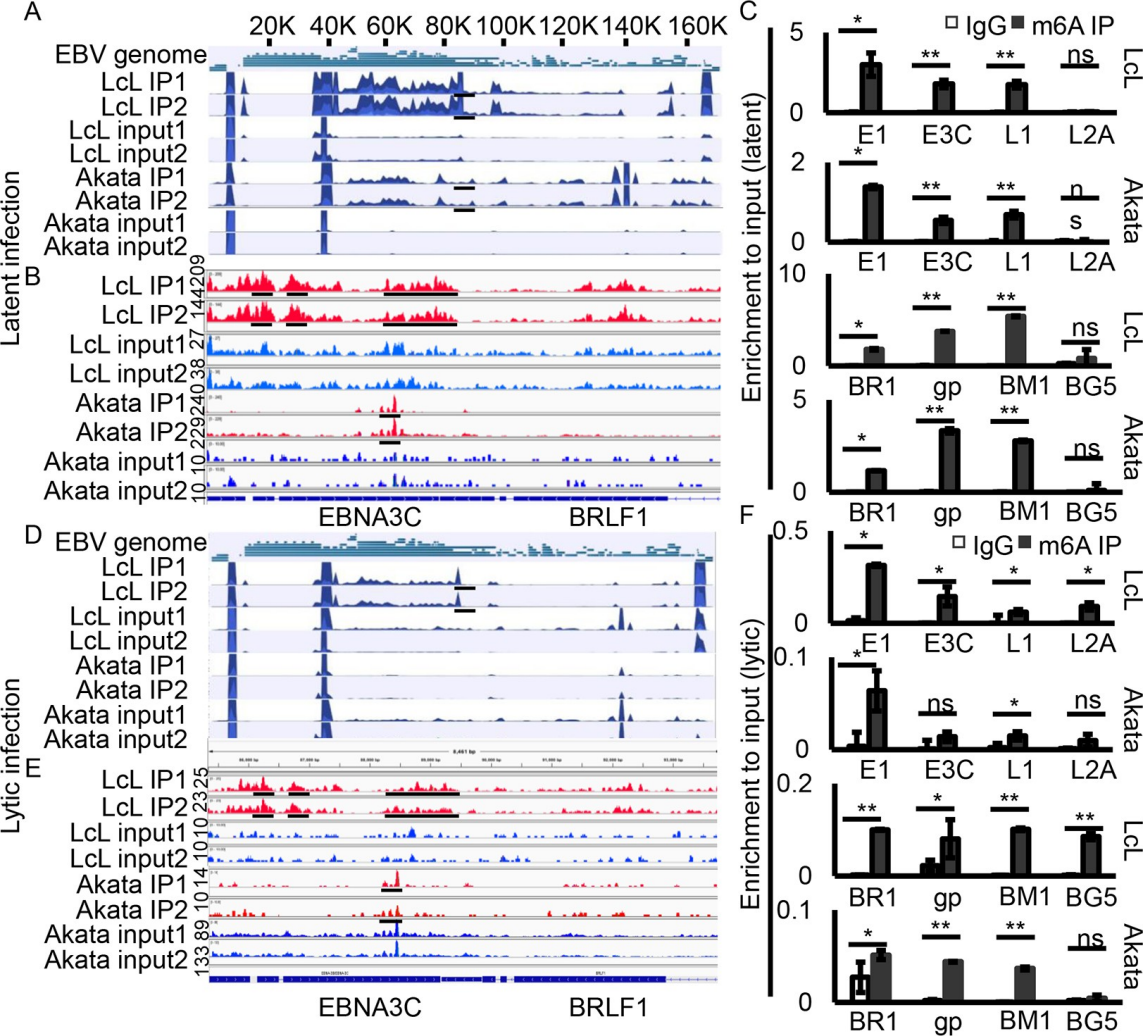
The EBV m6A epitranscriptome during viral latent and lytic infection. **(A, D)** Transcriptome-wide maps of EBV m6A IP reads, input reads and m6A peaks in LcLs and Akata cells with latent infection or lytically reactivated cells. **(B, E)** Enlarged regions of EBNA3C and BRLF1 from m6A modification sequencing data shows more detailed peaks. **(C, F)** Validation of m6A peaks in EBNA1 (E1), EBNA3C (E3C), LMP1 (L1), LMP2A (L2A), BRLF1 (BR1), gp350 (gp), BMRF1 (BM1) and BGLF5 (BG5) by MeRIP-qPCR. Fold enrichment was determined by calculating the fold change of IP to input Ct values. IgG precipitated RNA enrichment was used as the control. Experiments were independently repeated three times, and results are presented as mean±s.d. from the three experiments. The scale bar for the coverage tracks in Fig 1A and 1D is 1000. The others are labeled in the panel. “**” represents p-value <0.01; “*” represents p-value <0.05; “ns” represents no significance.

To determine the role of m6A modifications in the EBV lifecycle, we first examined the viral epitranscriptome in EBV transformed lymphoblastoid cell lines (LcLs) and EBV-positive Burkitt's lymphoma, Akata cells, using methylated RNA immunoprecipitation followed by sequencing (MeRIP-seq). Biological replicates of ribo-RNA deleted mRNA of each cell type were prepared for MeRIP-seq followed by peak calling using the MeTPeak package on both strands of the genome. We monitored expression of some lytic genes in LcLs through RNA-seq and RT-qPCR (S1A and S1D Fig). However, we found that there was a much lower level of lytic transcripts compared to latent gene transcripts based on the results of our realtime PCR assays. In addition, the results of biological replicates were also highly consistent. The m6A conserved peaks among LcLs and Akata cells were found in transcripts of both latent and lytic genes. The latent genes with m6A modifications include EBNA1, EBNA3A, EBNA3B, EBNA3C and LMP1 (Fig 1A and 1B, S1 and S2 Tables). M6A modification was also detected on the lytic transcripts including BRLF1, gp350 (BLLF1), DNA polymerase accessory subunit (BMRF1), as well as other lytic genes (Fig 1A and 1B, S1 and S2 Tables). The m6A peaks on EBNA1, EBNA3C, LMP1, BRLF1, gp350, and BMRF1 transcripts were confirmed by methylated RNA immunoprecipitation, reverse transcription, and quantitative real-time PCR (Fig 1C and 1F). The regions which encompassed the m6A peaks all displayed strong m6A enrichment compared to the IgG control. No m6A modification was detected on LMP2A transcripts from LcLs or Akata cells based on our qPCR analyses (Fig 1C). These results suggested that m6A modification was specifically distributed on both EBV latent and lytic transcripts.

### The m6A modification of EBV transcripts is decreased during lytic infection

We also investigated the levels at which the viral transcripts were modified during reactivation and induction of lytic replication. We detected m6A modification of EBV transcripts during lytic infection. TPA and Butyric acid were used to induce lytic reactivation. We observed a dramatic reduction of m6A modification of EBV transcripts both in reactivated LcLs and Akata cells (Fig 1D and 1E and S3 and S4 Tables). However, m6A peaks were still detected on specific transcripts encoded by latent genes, including EBNA3A, EBNA3B, EBNA3C, EBNA1, as well as BRLF1, gp350, and BMRF1 transcripts in lytically induced LcLs (Fig 1F). Notably, the fold enrichment in lytic infection was lower than that seen during latent infection and there was a typically 5-20-fold difference (Fig 1A, 1D, 1C and 1F). Additionally, the m6A modification signal was much lower in lytic Akata cells compared to lytic LcLs which ranged between 2–5 fold based on the MeRIP-seq data analysis and MeRIP-qPCR (Fig 1D and 1F). The comparatively high enrichment of m6A modification of latent viral transcripts and low enrichment of m6A modification during lytic infection suggested that m6A modification may play a role in latent gene expression and maintenance of EBV latent infection.

### Modification of EBV latent gene transcripts by m6A promotes stability of the mRNA

We identified an enrichment of m6A modifications of viral transcripts during EBV latent infection, and conversely a decrease in m6A modification of viral transcripts during lytic infection. Therefore, we hypothesized that m6A modification was important for latent antigen expression and stability in infected cells. One of the most important functions of m6A modification is its effects on mRNA stability. Therefore, we examined the role of m6A in regulating the mRNA stability expressed from several essential EBV genes.

**Fig 2:**
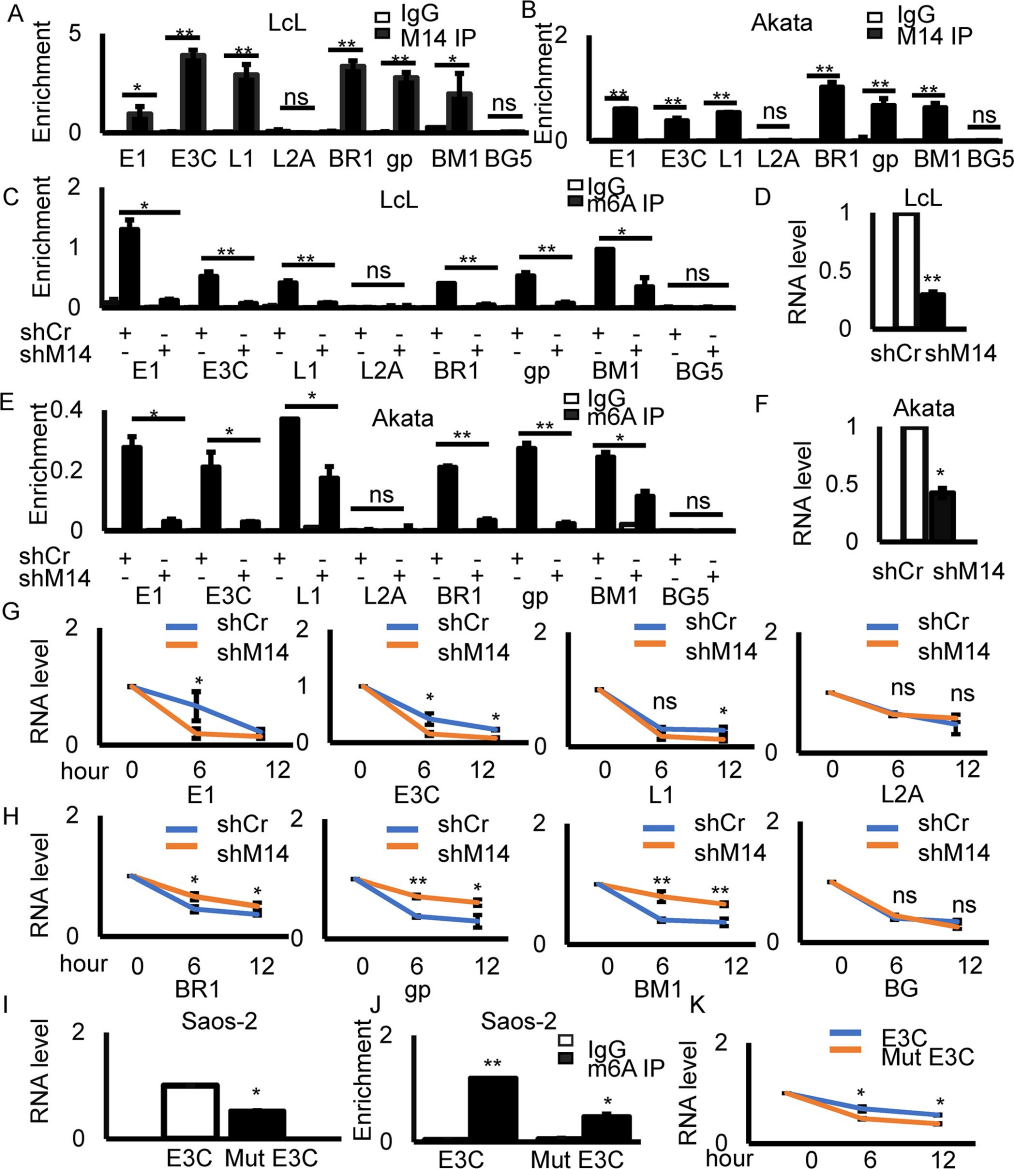
Modification of EBV latent gene transcripts by m6A promotes mRNA stability. **(A-B)** RIP using METTL14 antibody to detect the overall levels of METTL14 on viral genes in LcLs and Akata cells. Primers were designed for the indicated gene regions. **(C, E)** MeRIP using m6A antibody to detect overall levels of m6A on viral genes in LcLs and Akata cells with shCr or shMETTL14. Primers were designed for the indicated gene regions. **(D, F)** The RNA levels of METTL14 in shCr or shMETTL14 cells. **(G-H)** Quantification of levels of viral transcripts following treatment with actinomycin D in LcLs with shCr and sh METTL14. **(I)** RNA expression levels of wild-type and mutant EBNA3C. GAPDH was used as the reference control. **(J)** MeRIP using m6A antibody to detect the overall levels of m6A in the mutant and wild-type EBNA3C. **(K)** Actinomycin D was used to inhibit transcription for 6 and 12 hours and the levels of EBNA3C mRNA was determined. Experiments were independently repeated three times, and results are presented as mean±s.d. from the three experiments. “**” represents p-value <0.01; “*” represents p-value <0.05; “ns” represents no significance. UI: uninduced; IN: induced.

METTL14 is a major component of the methyltransferase complex required for m6A modification, and so we first determined binding of METTL14 to the transcripts modified with m6A. The target genes included 4 latent genes, EBNA1, EBNA3C, LMP1, LMP2A, and 4 lytic genes, BRLF1, gp350, BMRF1 and BGLF5. For input samples, similar results were observed that there were relatively higher levels of latent transcripts compared to lytic gene transcripts according to the results of our real-time PCR assays in latently infected LcLs. We showed the RNA levels of transcripts in LcL input samples in Fig 2A and 2C (S1B and S1C Fig), and also examined the corresponding DNA levels in IP samples and we did not find any DNA amplification before reverse transcription (S1D Fig). We found that there was a substantial increase in enrichment of m6A at EBNA1, EBNA3C, LMP1, BRLF1, gp350, and BMRF1, but not at LMP2A and BGLF5 with METTL14 immunoprecipitation (Fig 2A and 2B). These results were consistent with the MeRIP-seq and m6A-qPCR data described above.

To directly demonstrate the role of METTL14 in regulating these viral genes, we knocked down METTL14 followed by m6A RIP-qPCR. The results showed that m6A modification of EBNA1, EBNA3C, LMP1, BRLF1, gp350 and BMRF1 in the absence of METTL14 was dramatically decreased in both LcLs and Akata cells (Fig 2C–2F). We further investigated the viral mRNA stability after METTL14 knock down. The results showed that there were differences between the latent and lytic genes when METTL14 was knocked down. Consequently, the stability of the latent genes, including EBNA1, EBNA3C, and LMP1, was decreased in the absence of METTL14 (Fig 2G). However, the stability of lytic genes, including BRLF1, gp350, and BMRF1, was substantially increased in the absence of METTL14 as seen by the relative mRNA levels of the transcripts (Fig 2H). and the stability of LMP2A and BGLF5 were not affected (Fig 2G and 2H). The expression of METTL14 in the absence or presence of actinomycin D was monitored as a control. We found that the RNA level of METTL14 was decreased when the LcLs were treated with actinomycin D in both METTL14 knock-down cells and control cells (S2 Fig). These results demonstrated that METTL14 played an important role in m6A modification of viral transcripts and that m6A modification played a different role for latent and lytic genes in terms of their stability.

To investigate the role of specific m6A modification sites, we introduced 4 mutations to disrupt the DRACH motif (D = A/G/U, R = A/G, and H = A/C/U) [14] in the EBNA3C open reading frame (S12A Fig). The sites were selected according to the MeRIP-seq (Fig 1A and S1 Table) and qPCR data (Fig 1C). These residues are located at the amino-terminal region between G649 and C701 nucleotides where the highest enrichment of m6A modification within the EBNA3C transcript was identified. The mutations were designed to disrupt the DRACH motif where the adenosine is modified but would not change the underlying coding potential of the EBNA3C gene. The EBNA3Cwt and EBNA3Cmut constructs were transfected into Saos-2 cells and the expression of EBNA3C was detected using reverse transcription qPCR. We found that the levels of the EBNA3Cmut transcripts were lower than that of the wild-type EBNA3C (Fig 2I). The m6A IP-qPCR experiment showed that there was less m6A modification of the EBNA3C mutant transcript of greater than 2-fold compared to that of the wild-type EBNA3C (Fig 2J). To determine the effect of the mutations in EBNA3C, we used Actinomycin D to inhibit transcription for 6 and 12 hours. The RNA levels of EBNA3C was then measured. We found that there was a clear decrease in EBNA3C transcripts which contained the mutations (Fig 2K). These results demonstrated that m6A modification of the EBNA3C transcripts played an important role in EBNA3C mRNA expression levels and stability.

### Expression of the m6A methyltransferase METTL14, reader YTHDF2 and demethylase ALKBH5 is modulated in EBV-positive cells

We identified a large number of m6A modifications in EBV transcripts, and more specifically a different profile of m6A modification during latent and lytic infection. Therefore, we wanted to determine whether m6A related enzymes were regulated on EBV infection. We first performed RT-qPCR to detect the expression of m6A modification enzymes including METTL3, METTL14, YTHDF1, YTHDF2, YTHDF3, FTO and ALKBH5 in B cells, LcLs, Akata EBV negative and EBV-positive Akata cells. The RT-qPCR results showed that METTL14 was significantly up-regulated in EBV-positive LcLs and Akata cells (S3 Fig). YTHDF2 expression showed a significant decrease in LcLs and a modest decrease in Akata cells while ALKBH5 expression was slightly increased in LcLs and Akata cells (S3 Fig).

**Fig 3:**
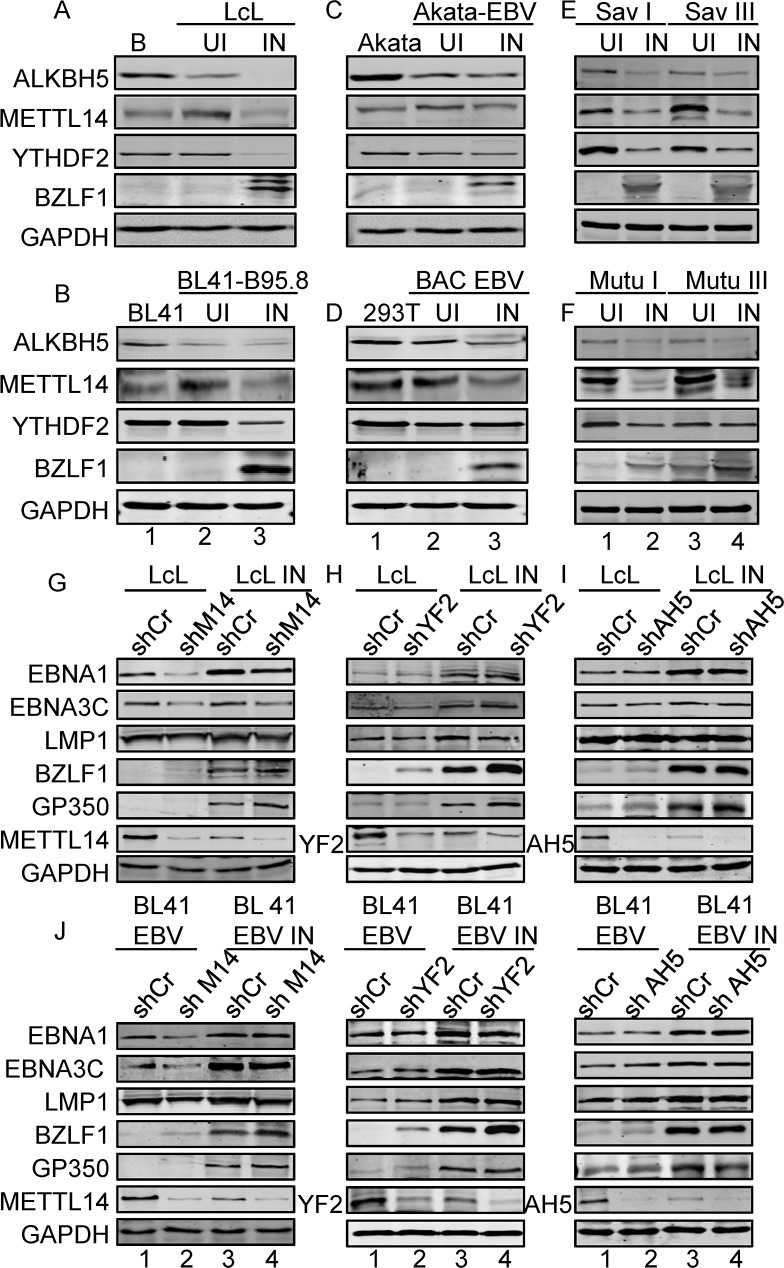
The expression of m6A enzymes in EBV infected cells and the role of METTL14, YTHDF2 and ALKBH5 on viral gene expression. **(A-D)** 10 million B cell, LcLs, Burkitt’s lymphoma (BL) cells BL41, Akata, EBV-positive BL41, Akata-EBV cells, 293T and 293T-BAC-EBV cells uninduced (Ul) were lysed with RIPA buffer and western blot analysis was performed with indicated antibodies. To reactivate EBV, TPA (20 ng/ml) and Butyric acid (BA, 2.5 mM) were used to treat the cells for 48 hours (IN). **(E-F)** 10 million SavI, SavIII, MutuI and Mutu III cells were lysed with RIPA buffer and western blot analysis was performed with indicated antibodies. To reactivate EBV, TPA (20 ng/ml) and Butyric acid (BA, 2.5 mM) were used to treat the cells for 48 hours. **(G-I)** 5 million LcL shCr, LcL shMETTL14 (shM14), LcL shYTHDF2 (shYF2), LcL shALKBH5 (shAH5) cells were collected, lysed and subjected to western blot with indicated antibodies. **(J-L)** 5 million BL41-EBV shCr, BL41-EBV shMETTL14, BL41-EBV shYTHDF2, BL41-EBV shALKBH5 cells were collected, lysed and subjected to western blot with the indicated antibodies. UI: uninduced with drugs; IN: induced with drugs.

We further examined the protein levels of METTL14, YTHDF2, and ALKBH5 since there were differences in their RNA expression. EBV negative B cells, Akata EBV-, BL41 and HEK293T cells, and latently infected LcLs, BL41-EBV and HEK293T-BAC-EBV cells were used for analysis of the protein levels by Western blot. The expression of the demethylase ALKBH5 was significantly decreased while the m6A methyltransferase METTL14 was dramatically increased in EBV-positive LcLs, BL41-B95.8 and there is a modest increase in Akata and 293T-BAC-EBV cells (Fig 3A–3D, lane 1–2 and S4A–S4D Fig). These results suggested that the m6A methyltransferase and m6A modification are required for EBV latent infection. Further, the expression of METTL14, YTHDF2 and ALKBH5 were also detected during lytic reactivation of infected cells. The data clearly showed that expression of METTL14, YTHDF2 and ALKBH5 was down-regulated when EBV-positive cells were induced to lytic reactivation (Fig 3A–3D, lane 3, and S4A–S4D Fig).

The expression of METTL14, YTHDF2 and ALKBH5 was also monitored in several other EBV-infected cells. Latent Sav I, Sav III, Mutu I, and Mutu III as well as reactivated Sav I, Sav III, Mutu I, and Mutu III cells were used for analysis of the protein levels. BZLF1 was detected by western blot to confirm reactivation of EBV. We showed that METTL14 expression was increased in latency EBV III cells (Mutu III and Sav III), compared to latency I cells (Mutu I and Sav I) (Fig 3E and 3F, lane 1 and 3). In addition, the expression levels of reader YTHDF2 and the demethylase ALKBH5 were decreased in latency III EBV cells compared to latency I EBV cells (Fig 3E and 3F, lane 1 and 3). The expression levels of METTL14, YTHDF2 and ALKBH5 in lytically reactivated EBV-positive Sav I, Sav III, Mutu I and Mutu III cells were consistently down-regulated (Fig 3E and 3F, lane 2 and 4). This result suggests that essential viral antigens expressed during latency III may promote the expression of METTL14 and that its expression is reversed during lytic reactivation.

METTL14-mediated m6A modification regulated the stability of EBV mRNA. To further examine the role of METTL14, YTHDF2 and ALKBH5 in regulating the expression of EBV proteins, METTL14, YTHDF2 and ALKBH5 were knocked down with lentivirus carrying shRNA in EBV-positive Burkitt lymphoma cells and LcLs. Knock-down of METTL14 led to a decrease in latent viral gene expression, including EBNA1, EBNA3C and LMP1 (Fig 3G and 3J, S4G, and S4J Fig). Expression levels of two lytic genes, BZLF1 (immediately early) and GP350 (late) were modestly induced by knock-down of METTL14 in EBV-positive cells (Fig 3G and 3J), and EBNA1, EBNA3C and LMP1 expression levels did not show any noticeable change in the YTHDF2 and ALKBH5 knock-down cells (Fig 3H–3L, S4H–S4L Fig). However, BZLF1 expression was increased in YTHDF2 knock-down cells (Fig 3H and 3K).

EBV reactivation was induced by TPA and Butyric acid, and the expression of viral genes was monitored. In contrast to latently infected cells, expression of the viral proteins was seen to be significantly increased in reactivated cells (Fig 3G–3L). Notably, latent gene expression was lower in METTL14 knock-down cells, but the lytic genes BZLF1 and GP350 were increased in METTL14 knock-down cells by at least 2-fold (Fig 3G and 3J). These two lytic genes were also increased in the YTHDF2 knock-down cells (Fig 3H and 3K). However, there were little or no obvious effects of ALKBH5 knock-down on viral antigen expression (Fig 3I and 3L). We investigated the function of m6A modification in the virus lytic cycle progression and the production of infectious virions through knocking down METTL14 in LcLs. We found that knock-down of METTL14 alone will not lead to robust reactivation. However, knock-down of METTL14 will promote virion production when the virus entered the lytic cycle (S5A Fig). These results demonstrated that METTL14 played a role in promoting EBV antigen expression during latent infection as well as lytic infection. Since METTL3 and METTL14 work as a methyltransferase complex. We further monitored the binding of METTL3 on the m6A sites of viral transcripts in LcLs and the results showed that METTL3 also bound to the m6A sites (S5B Fig). We then investigated the effects of METTL3 on viral gene expression and we found that METTL3 knock-down showed a similar consequence for EBV protein expression as the METTL14 knock-down (S5C Fig). Meclofenamic acid is reported to be a specific demethylation inhibitor [15]. To rule out any possible effects due to ALKBH5 shRNA artifacts, we treated LcLs with meclofenamic acid to support the results of the ALKBH5 knock-down. We found that the demethylation inhibitor showed similar effects as the ALKBH5 knock-down on viral gene expression (S5D Fig).

### EBNA3C regulates METTL14 expression at the transcription level

We further investigated which antigen of EBV was responsible for up-regulation of METTL14. We transfected Myc-tagged EBNA2, EBNA3C, LMP1, LMP2A and LMP2B in Saos-2 cells (S6A–S6G Fig). EBNA1 was not included as we observed a dramatic increase in METTL14 levels in type III EBV-positive latent cell lines compared to that of type I EBV-positive latent cell lines (Fig 3E and 3F). 48 hours later, cells were collected and lysed. METTL14 and the transfected viral proteins were detected by western blot analyses. We found that EBNA3C was able to up-regulate METTL14 expression by approximately 2.5-fold (S6G Fig). We repeatedly found that METTL14 was up-regulated in LcLs compared to EBV negative B cells. In addition, LMP1 upregulated METTL14 expression by approximately 24% at the protein level (S6B Fig). We also showed that METTL14 expression levels were decreased by about 2-fold in EBNA3C knock-down LcLs (S6H Fig), and further confirmed these results in EBNA3C stably expressed B cell lines BJAB7 and BJAB10 cells. METTL14 expression levels were increased in the presence of EBNA3C, when compared to EBNA3C negative B cells and was based on the levels of EBNA3C expressed (S6I Fig).

We also monitored the expression of METTL14 at the transcription level in EBV-positive and EBNA3C expressing cells. EBNA3C expression can up-regulate METTL14 transcription in BJAB7 and BJAB10 cells which stably expressed EBNA3C (S6J Fig). We found that the RNA levels of METTL14 were also increased in EBV-positive cells and knock-down of EBNA3C in LcLs led to decreased expression of METTL14 (S6K Fig). These results demonstrated that EBV infection, and specifically, EBNA3C expression can up-regulate METTL14 transcription. Further, we generated the METTL14 promoter-driven luciferase reporter system to determine whether EBNA3C was able to up-regulate METTL14 expression through activation of the METTL14 promoter. The reporter construct containing the METTL14 promoter and a dose-dependent increase of the Myc-EBNA3C expression vector was transfected into HEK293 or Saos-2 cells. Meanwhile, the thymidine kinase promoter-Renilla luciferase reporter plasmid (pRL-TK) was additionally transfected and used as a control for transfection efficiency. The results of the luciferase assay suggested that the METTL14 promoter activity was dramatically increased by EBNA3C in a dose-dependent manner (S6L–S6M Fig). These results demonstrated that the essential EBV latent protein EBNA3C can up-regulate the methyltransferase METTL14 transcription through activation of the METTL14 promoter.

### METTL14 is stabilized by EBNA3C

**Fig 4:**
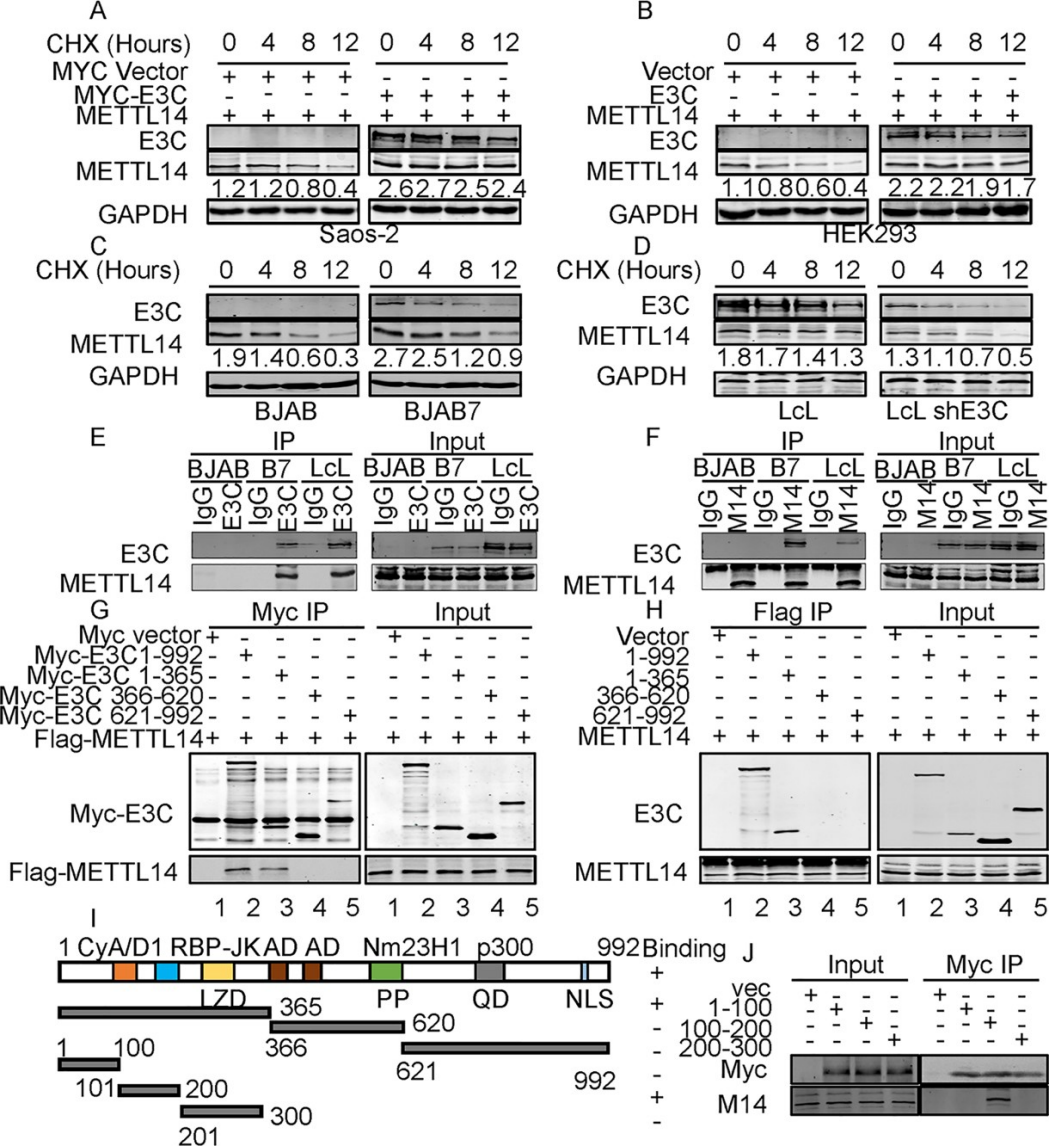
EBNA3C stabilizes METTL14 through interaction in human cells. **(A, B)** 10 million Saos-2 or HEK293 cells were co-transfected with control plasmids, Myc-E3C, and Flag-METTL14 expression plasmids. 36 hours later, cells were treated with 40 μg/ml cycloheximide (CHX) for 0, 4, 8, 12 hours, and then cells were collected, lysed and subjected to western blot with indicated antibodies. (**C, D)** BJAB, BJAB7, LcL shCr, and LcL shE3C cells were treated with 40 μg/ml cycloheximide (CHX) for 0, 4, 8, 12 hours, and then cells were collected, lysed and subjected to western blot with indicated antibodies. **(E, F)** 30 million BJAB, BJAB7, and LcLs were lysed with RIPA buffer and co-immunoprecipitation was performed with EBNA3C (E) or METTL14 (F, M14) specific antibody. Immunoprecipitated samples were resolved by 10% SDS-PAGE and endogenous EBNA3C and METTL14 were detected by the specific antibodies. **(G, H)** 30 million HEK293 cells were transfected with indicated plasmids and lysed with RIPA buffer 48 hours later, and co-immunoprecipitation was performed with Myc or Flag antibody. Immunoprecipitated samples were resolved by 10% SDS-PAGE and proteins were detected by the specific antibodies. **(I)** The schematic diagram shows the 3 main regions of EBNA3C. NLS, nuclear localization signal; LZD, leucine zipper domain; AD, acidic domains; PP, Proline-rich; QP, glutamine-proline-rich. **(J)** Myc-tagged specific regions of EBNA3C (1-100aa, 100-200aa, or 200-300aa) were co-transfected into 10 million HEK293 cells with Flag-tagged METTL14. Co-immunoprecipitation was performed with Myc antibodies. EBNA3C and METTL14 were detected by the specific antibodies.

We have shown that METTL14 transcripts and protein levels were significantly up-regulated by EBNA3C. To demonstrate whether EBNA3C can regulate METTL14 at the protein level, we initially performed protein stability assays by expressing METTL14 with or without EBNA3C in Saos-2 and HEK293 cells. Cells were treated with cycloheximide (CHX) for up to 12 hours. The protein level of METTL14 was decreased dramatically in the absence of EBNA3C (Fig 4A and 4B). The results demonstrated that METTL14 was stabilized on expression of EBNA3C (Fig 4A and 4B). We further validated the results by treating BJAB, BJAB7, LcL-shCr and LcL-shE3C cells with CHX. The results showed that the stability of METTL14 was significantly enhanced in the presence of EBNA3C compared to a loss of up to 63% of the METTL14 signal at 12 hours after cycloheximide treatment in EBNA3C-negative cells (y4C and 4D).

We further examined whether EBNA3C can interact directly with METTL14. Co-immunoprecipitation (Co-IP) assays were performed in B-cells with physiologic expression of EBNA3C. To determine the association in B-cell lines, BJAB, EBNA3C stably expressing BJAB (BJAB7) and EBV-transformed (LcL) cells were used for the Co-IP experiment. We performed IP using an EBNA3C antibody or METTL14 specific antibody to immunoprecipitate the target proteins followed by western blot analyses. The Co-IP results showed that endogenous EBNA3C can physically associate with METTL14 and more importantly in EBV-transformed lymphoblastoid cell lines (Fig 4E and 4F).

To determine the domain of EBNA3C which can specifically interact with METTL14, Myc-tagged full length and specific regions of EBNA3C (1-365aa, 366-620aa or 621-992aa) were co-transfected into HEK293 cells with Flag-tagged METTL14 (Fig 4I). The targeted protein was immunoprecipitated with anti-Myc or anti-Flag antibody, respectively. The results demonstrated that METTL14 interacted with an amino terminal region of EBNA3C (1-365aa) as well as with the full-length EBNA3C protein (1-992aa) (Fig 4G and 4H). These results showed that EBNA3C amino acid residues 1-365aa were responsible for the interaction between EBNA3C and METTL14 (Fig 4G and 4H). Plasmids with EBNA3C truncations were also transfected in HEK293 cells without Flag-METTL14 plasmid. IP was done and the results showed that there is no non-specific binding to Flag protein (S7 Fig). To further determine the exact domain of EBNA3C which can specifically interact with METTL14, Myc-tagged specific regions of EBNA3C (1-100aa, 100-200aa, or 200-300aa) which delineated specific motifs in the amino terminal region were co-transfected into HEK293 cells with Flag-tagged METTL14. Co-immunoprecipitation was performed with Myc antibodies. The results suggested that the region located within residues 100-200aa of EBNA3C containing the interaction domain for the transcription repressor RBP-JK was responsible for its interaction with METTL14 (Fig 4J).

To determine whether the EBNA3C residues EBNA3C100-200 or EBNA3CΔ100–200 (EBNA3C without 100-200aa residues) affect the protein expression, protein stabilities and the oncogene function of METTL14, we expressed EBNA3C100-200, EBNA3CΔ100–200, or EBNA3C in HEK293 cells. We found that only the full-length EBNA3C can upregulate the expression of METTL14. Neither the EBNA3C100-200 alone nor EBNA3C lacking the 100–200 residues up-regulated METTL14 expression (S8A Fig). We also monitored the effects of EBNA3C100-200 and EBNA3CΔ100–200 on METTL14 protein stability. We found that neither the EBNA3C100-200 alone nor EBNA3C lacking the 100–200 residues promoted the stability of METTL14 (S8B and S8C Fig).

### EBNA3C colocalized with METTL14 in EBV-transformed LcLs and Post-transplant Lymphoproliferative patient tumor tissues

We have shown that EBNA3C binds to METTL14, so it is expected that these two proteins can coexist in the same cellular compartment and colocalize with each other in cells. To determine the co-localization of EBNA3C and METTL14, Saos-2 and HEK293 cells were initially transfected with plasmids expressing Myc-tagged EBNA3C, and the cellular localization of EBNA3C and METTL14 was monitored by fluorescence microscopy. In the absence of EBNA3C, the METTL14 proteins were distributed around the nucleus as seen with some punctate dots (S9A Fig). In cells transfected with Myc-EBNA3C, the merged yellow fluorescence demonstrated that EBNA3C co-localized with METTL14 in Saos-2 and HEK293 cells (S9B–S9D Fig).

**Fig 5:**
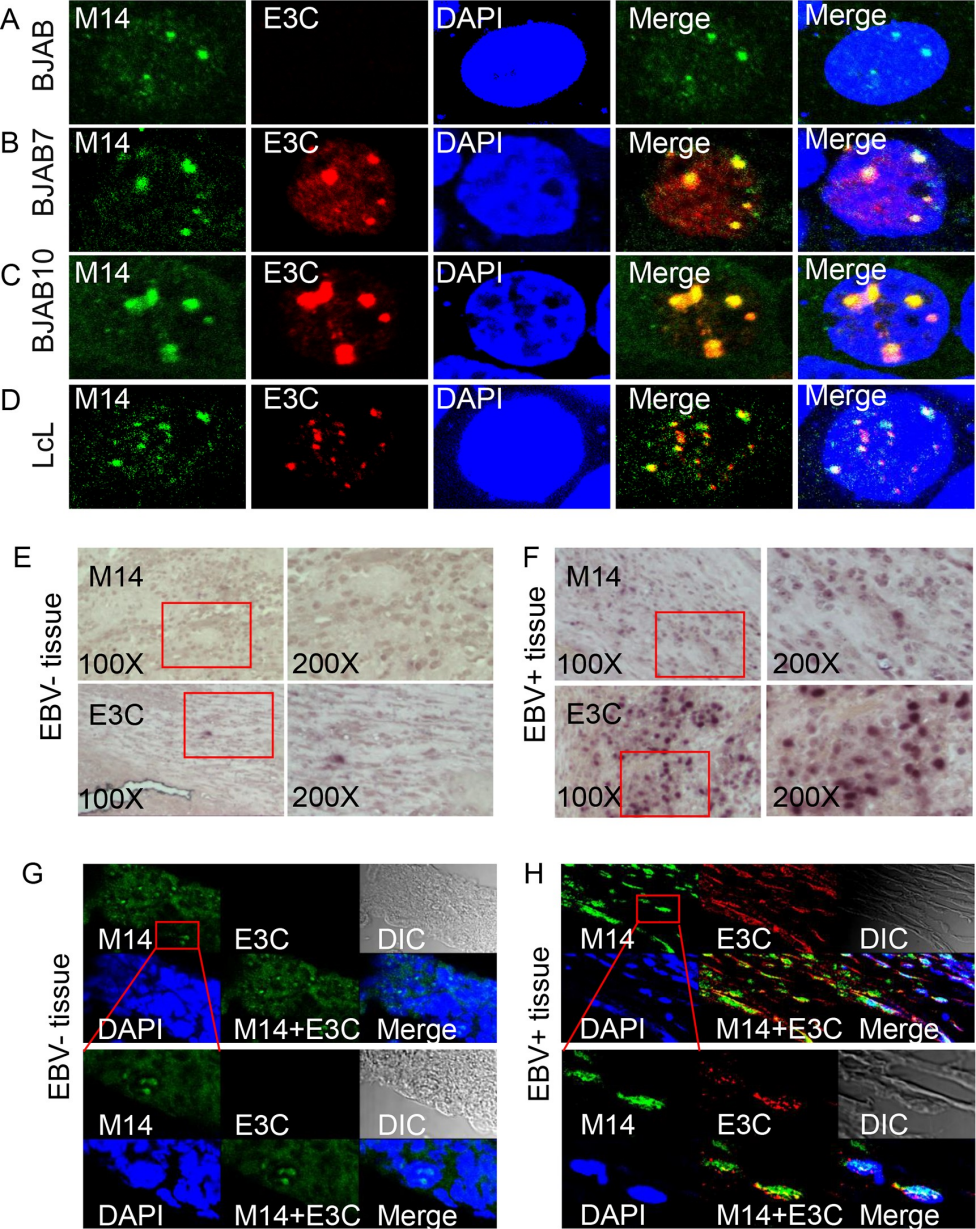
EBNA3C colocalizes with METTL14. **(A-D)** BJAB, BJAB7, and LcLs were plated on slides and cells were fixed with 4% PFA, and stained with specific antibodies. EBNA3C was detected by mouse anti-EBNA3C (A10) antibodies followed by the secondary anti-mouse Alexa Fluor 594. METTL14 were detected by rabbit anti-METTL14 antibodies, followed by anti-rabbit Alexa Fluor 488 as the secondary antibody. The nuclei were subsequently stained with DAPI, and the images were captured using an Olympus Fluoview confocal microscope. The results shown are representative of three independent experiments. **(E, F).** Representative images (left, 200X; right, 400X) of immunohistochemistry staining against METTL14 or EBNA3C in EBV negative tissue or positive PTLDs tumor tissue. **(G, H)** Immunofluorescent analysis of METTL14 or EBNA3C in EBV negative tissue or positive PTLDs tumor tissue.

To further determine the co-localization of EBNA3C and METTL14 proteins in EBV-positive B-cells, immune-fluorescence assays were performed using specific antibodies against EBNA3C and METTL14 in order to examine the endogenous expression in different B-cell lines. The results corroborated the above results that EBNA3C co-localized with METTL14 in BJAB cells that can stably express EBNA3C, as well as in EBV-transformed LcLs (Fig 5A–5D). This was consistent with the above results and further suggested that EBNA3C associated with METTL14 in nuclear complexes.

We further investigated the expression and localization of METTL14 in EBV-negative and positive human tumor tissues. Immunohistochemistry for METTL14 expression showed that it was clearly up-regulated in EBV-positive Post-transplant lymphoproliferative disorders (PTLDs) tumor tissues and that EBNA3C signals were also clearly detected in the EBV-positive tissues compared to EBV negative tissues (Fig 5, compare panels 5E and 5F). We supported our co-localization data for METTL14 and EBNA3C in the PTLD tumor tissues with immunofluorescence assays. The results showed that EBNA3C co-localized with METTL14 in PTLD tumor tissues (Fig 5, compare panel 5G and 5H).

### METTL14 induces proliferation and colony formation of EBV positive cells

**Fig 6:**
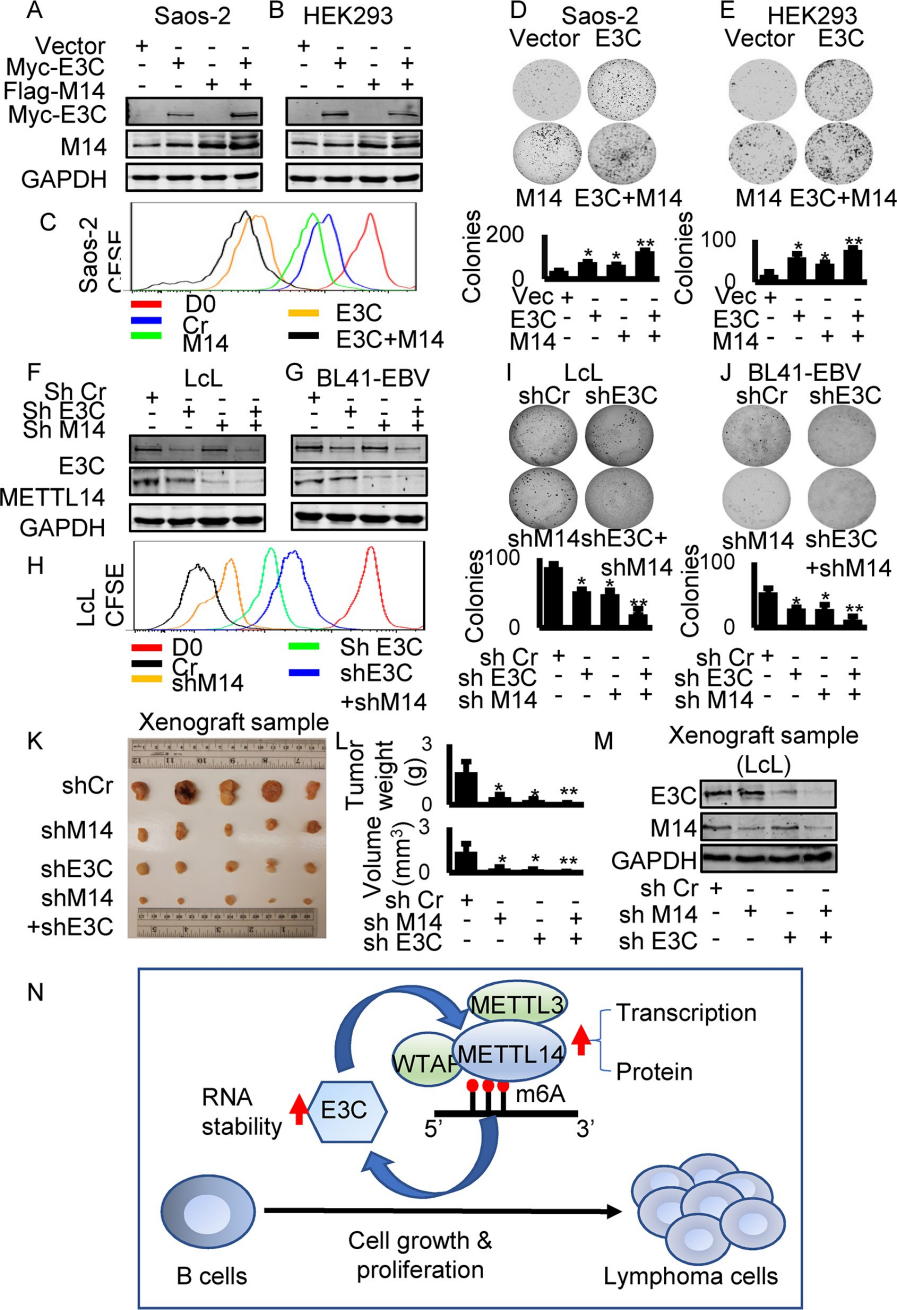
METTL14 facilitates cell growth and proliferation in EBV infected cells. **(A-E)** HEK293 or Saos-2 cells were transfected with control vector, Myc-EBNA3C, Flag-METTL14 or Myc-EBNA3C plus Flag-METTL14 and allowed to grow in DMEM supplemented with 1mg/ml G418. After two weeks of selection, 10^5^ cells were seeded in 6-well plate and allowed to grow for 5 days. The cells were stained with 0.005% Crystal Violet overnight and scanned by PhosphorImager and the area of the colonies measured by using Image J software. The same number of G418 selected cells were seeded to 6-well plates, collected and lysed in lysis buffer after 5 days culture. The lysate was subjected to western blot with indicated antibodies. 1X10^5^ cells were stained with 5μM CFSE for 10 minutes at 37°C. The cells were washed, cultured, and harvested after culturing for 3 days. Flow cytometry was used to analyze CFSE-labeled cells. (**F-H)** LcL shCr, LcL shMETTL14, LcL shEBNA3C, and LcL shMETTL14 plus shEBNA3C cells were established with lentivirus-mediated knock-down. Western blot was performed to confirm the expression of EBNA3C or METTL14. 1X10^5^ cells were stained with 5μM CFSE for 10 minutes at 37°C. Cells were washed, cultured, and harvested after culturing for 3 days. Flow cytometry was used to analyze CFSE-labeled cells. (I) LcL shCr, LcL shMETTL14, LcL shEBNA3C, LcL shMETTL14 plus shEBNA3C were assessed for their ability to promote colony formation using the soft agar assays. Photomicrograph of representative colonies from three independent experiments is shown. (J) BL41-EBV shCr, BL41-EBV shMETTL14, BL41-EBV shEBNA3C, BL41-EBV shMETTL14 plus shEBNA3C were assessed for their ability to promote colony formation using the soft agar assays. Photomicrograph of representative colonies from three independent experiments is shown. **(K-L)** 6 million LcL shCr, LcL shMETTL14, LcL shEBNA3C, and LcL shMETTL14 plus shEBNA3C were subcutaneously injected into NOD-SCID mice to assess the effect of knocking down METTL14 or EBNA3C on tumor growth *in vivo*. 3 weeks later, mice were sacrificed and the weight and size of the tumor were measured. **(M)** Tumor tissues were homogenized and subjected to western blot analysis. **(N)** The interaction between EBV antigen EBNA3C and METTL14. EBV transcripts are modified by METTL14 and its partners. m6A modification promotes EBV latent gene expression which is critical to EBV-induced oncogenesis. The essential EBV latent antigen EBNA3C promotes METTL14 transcription and protein stability through direct interaction during EBV latent infection. The interaction feedback loop between EBV antigen EBNA3C and METTL14 promotes EBV-mediated cell growth and proliferation.

To examine the effects of METTL14 on cell proliferation, Saos-2 and HEK293 cells were transfected with expression of EBNA3C and METTL14 and selected with G418 for two weeks. The expression of EBNA3C and METTL14 was confirmed by western blot (Fig 6A and 6B). We monitored cell proliferation by expressing EBNA3C or METTL14 using CFSE staining assays. The results showed that both EBNA3C and METTL14 expression can promote enhanced cell growth (Fig 6C). We further investigated the growth of colonies in monolayer cultures. Cells were seeded into 6-well plates and allowed to grow for 5 days to monitor formation of colonies. A significant increase in colony numbers was observed when EBNA3C and METTL14 were co-transfected compared to those transfected with only EBNA3C or METTL14 in both cell types (Fig 6D and 6E). We performed overexpression of METTL14, EBNA3C, and co-overexpression of EBNA3C and METTL14 in BJAB cells. And again, we found similar phenotypes of overexpression of METTL14 and EBNA3C in BJAB cells (S10 Fig). These results demonstrated that METTL14 can promote growth of cells and that this ability was further enhanced by EBNA3C. We further monitored the role of METTL14 in cell growth in the context of EBV transformed LcLs. We performed CFSE staining and soft agar assays with LcL shCr, LcL shEBNA3C, LcL shMETTL14 and LcL shEBNA3C plus shMETTL14. Knock-down of EBNA3C and METTL14 was confirmed by western blot (Fig 6F). The results showed that knock-down of EBNA3C or METTL14 inhibited the ability of LcLs to form colonies in the soft agar assay (Fig 6H and 6I). Furthermore, we observed the most prominent inhibition of colonies in LcLs with both EBNA3C and METTL14 knock-down (Fig 6H and 6I). In support of these results, we observed a similar pattern of cell growth in Burkitt lymphoma BL41 cells with these genes knocked down (Fig 6G and 6J). The results demonstrated that METTL14 enhanced cell proliferation and growth of colonies in EBV transformed cells by cooperating with EBNA3C.

To investigate the functional role of METTL14 in EBV-associated tumorigenesis, we probed the effects of its knock-down in a xeno-transplant model in vivo. LcL shCr, LcL shMETTL14, LcL shEBNA3C and LcL shMETTL14 plus shEBNA3C were subcutaneously injected into NOD-SCID mice to assess the effect of knock-down METTL14 or EBNA3C on tumor growth. The results demonstrated that knock-down of METTL14 or EBNA3C led to a dramatic slowing of tumor growth compared to the control group (Fig 6K–6M and S11 Fig). Importantly, knock-down of both METTL14 and EBNA3C together led to the most dramatic decrease in tumor weight and volume (Fig 6K–6M). Western blots to show levels of METTL14 and EBNA3C showed that METTL14 was knocked down greater than 50% and EBNA3C greater than 75% of the endogenous signals in the LcLs (Fig 6M).

We also investigated the functional role of specific m6A modification sites on EBNA3C for cell growth. We transfected the wild-type or EBNA3C mutant at the m6A sites into BJAB (EBV negative cells) and Raji cells (EBV-positive but without EBNA3C expression) [16]. Cell growth was monitored by CFSE staining and Soft agar assays. The results showed that mutant EBNA3C had a compromised capacity to promote cell growth when compared to the wild-type EBNA3C in both BJAB and Raji cells (S12B–S12E Fig). These results confirmed a critical role for m6A modification of EBNA3C through regulation of METTL14 in driving the oncogenic phenotype. Finally, we investigated the function of EBNA3C100-200 and EBNA3CΔ100–200 in the oncogene function of METTL14 though Soft agar and CFSE staining assays. We found that neither the EBNA3C100-200 alone nor EBNA3C lacking the 100–200 residues promoted METTL14-mediated cell growth compared to full-length EBNA3C (S8D–S8G Fig).

## Discussion

Modification of RNA by m6A functions extensively in cellular processes linked to RNA metabolism. These include mRNA stability, translation, splicing and RNA transport [17]. This modification is also involved in the self-renewal of cancer stem cells, promotion of cancer cell proliferation, and resistance to radiotherapy or chemotherapy [17]. Recent studies have provided clues as to understanding the roles of m6A modification during virus infection, however, the function of m6A modification in tumor virus-mediated oncogenesis is completely unknown and not previously explored. We have now mapped the m6A modification of viral transcripts during latent and lytic infection of EBV. We showed that m6A modification can play a major role in promoting latent gene expression and also repression of lytic gene expression. We also show that the level of methyltransferase METTL14 was up-regulated by the essential EBV latent antigen EBNA3C. Furthermore, EBNA3C promoted cell growth and proliferation by co-operating with METTL14. We now establish a novel link between regulation of the EBV epitranscriptome and EBV-mediated oncogenesis (Fig 6N).

EBV latent infection allows the virus to persist in a mostly dormant state for the lifetime of the host and is associated with numerous cancers [18]. EBV protein expression in latency III potently drives B-cells to immortalization through regulation of cell growth, and promotion of cell survival. Increased expression of c-Myc can also be induced by EBV latent antigens, such as EBNA2 [19], LMP2A [20], and EBNA3C [21]. The ubiquitin ligase SCFSKP2, cyclin D1, cyclin A, c-Myc, MDM2, p53, CHK2, E2F1, and E2F6 are all directly regulated by EBNA3C [22–25]. Additionally, EBV latent proteins can play an essential role in epigenetic deregulation during B-cell lymphomagenesis [26,27]. EBV latent antigens are the major contributors to EBV-associated malignancies. Therefore, decreased expression of EBV latent antigens will result in attenuation of EBV-mediated tumorigenesis.

Modification of RNA by m6A is catalyzed by a methyltransferase complex composed of METTL3, METTL14, and WTAP [17], and mRNAs containing m6A modification can be recognized by YTH domain family members, YTHDF1, YTHDF2 and YTHDF3 [28]. The consequence of m6A modification differs and depends on being recognized by the readers, and the position of the m6A modification [29]. YTHDF1 is also known to enhance translation of mRNA [30]. YTHDF2 is now known to induce mRNA degradation [31], and YTHDF3 co-operates with either YTHDF1 or YTHDF2 to promote translation or degradation of transcripts, respectively [32]. More recently another family of m6A readers, insulin-like growth factor 2 mRNA-binding proteins (IGF2BPs; including IGF2BP1/2/3) have been shown to up-regulate mRNA stability [33]. IGF2BPs belong to a conserved family of single-stranded RNA binding proteins [33], and can recognize m6A modification to enhance mRNA stability and translation [33]. IGF2BPs and YTHDF2 show a different distribution on the transcripts and recognize m6A modification with different motif sequences [33]. Nevertheless, new m6A readers are emerging and there must be more unrevealed readers to be explored. We found that m6A modifications are distributed on both latent and lytic gene transcripts. M6A modification at the viral latent and lytic gene transcripts resulted in different outcomes. Modification of latent transcripts promoted stability while it had a negative role on stability of lytic transcripts. This difference may be due to the different readers responsible for recognition of the modification. Additionally, the enhanced stability of the latent transcripts, such as EBNA3C, had a direct effect on promoting cell growth and proliferation.

METTL3-METTL14-WTAP-mediated m6A modification is associated with cancer [5] and the relationship between m6A and cancer has not been fully explored. Silencing of METTL3 led to P53 signaling pathway enrichment in HepG2 cells [5], and knock-down of METTL3 or METTL14 promoted glioblastoma stem cell growth and tumorigenesis [34]. METTL14 is highly expressed in normal hematopoietic stem/progenitor cells and acute myeloid leukemia cells [35]. METTL14 is required for myeloid leukemia cell survival and proliferation [35]. We found that METTL14 expression was dramatically up-regulated in EBV-infected cells, and EBNA3C positive cells. We also showed that METTL14 promoted growth and proliferation of these cells. Importantly, we showed upregulation of METTL14 in EBV-positive PTLDs and knock-down of METTL14 resulted in a decreased tumorigenic activity of EBV-transformed cells in the xenograft animal model systems.

Studies in KSHV revealed that blocking m6A inhibits splicing of the pre-mRNA encoding replication transcription activator (RTA), a key KSHV lytic switch protein, and halts viral lytic replication [36]. Knockdown of YTHDF1, YTHDC1 or YTHDC2 had no significant or consistent effect on viral lytic replication [12]. Knock-down of YTHDF2 led to a four-fold increase in KSHV production and an increase in RTA, ORF57, ORF-K8 and ORF65 lytic transcripts through an increase of the half-life of KSHV transcripts. Another study found that depletion of the m6A machinery had differential pro- and anti-viral impacts on viral gene expression depending on the cell-type analyzed [37]. Here we focused on METTL14, a component of methyltransferase complex and found that METTL14 can promote increased levels of the latent oncogene EBNA3C. METTL3-METTL14 mediated m6A modification can negatively regulate EBV lytic gene expression, but knock-down METTL14 cannot drive EBV into full-blown lytic reactivation from latency. Although we focused on the relationship between m6A modification and EBV-mediated tumorigenesis, the effects of METTL3-METTL14 mediated m6A modification on lytic gene expression is an interesting topic for further study. METTL14-mediated m6A modification can negatively regulate mRNA stability of some lytic genes according to our initial results. Correspondingly, METTL14 expression is dramatically downregulated during the EBV lytic infection cycle. These results suggest that m6A modification is likely not absolutely a necessary requirement for EBV lytic reactivation. The detailed mechanism related to EBV reactivation and m6A modification will need to be further explored.

In this study, we have now shown for the first time that the host RNA m6A modification machinery can be targeted and manipulated specifically by a viral-encoded oncoprotein. This modulated latent and lytic viral gene expression enhancing the pro-oncogenic potential of EBV. The study now provides a deeper understanding of specific interactions between a potent oncogenic virus and host cells at the level of the viral epitranscriptome, and further suggests a critical role for viral mRNA modification in driving the oncogenic process. Furthermore, targeting METTL14 may be a critical strategy for controlling EBV-associated cancers.

## Materials and methods

### Ethics statement

The University of Pennsylvania Institutional Animal Care and Use Committee reviewed and approved all the animal experiments (protocol 804549). All the experiments were carried out in accordance with the Laboratory Animal Welfare guidelines for the Care and Use provided by National Institutes of Health. The University of Pennsylvania CFAR Immunology Core provided B-cells from deidentified humans. The CFAR Immunology Core maintains an Institutional Review Board (IRB)-approved protocol in which Declaration of Helsinki protocols are strictly followed. Every donor gave written and informed consent at the CFAR Immunology Core. All patients provided written informed consent.

### Cell lines, Plasmids, and antibodies

The plasmid pA3M-EBNA3C and the LcLs, Akata, and BJAB cell lines, have been described previously [4]. LcLs were generated in our laboratory [4]. BJAB, BL41, Akata, Akata-EBV, and BL41-B95.8 cell lines were kindly provided by Elliott Kieff (Harvard Medical School, Boston, MA). Saos-2 (human osteosarcoma cell line) and HEK-293 (human embryonic kidney cell line) were provided by Jon Aster (Brigham and Woman's Hospital, Boston, MA). Sav I and Sav III cell lines, Mutu I and Mutu III cell lines were kindly provided by Dr. Paul M. Lieberman (The Wistar Institute, Philadelphia, PA). Antibodies against Myc (9E10) and EBNA3C antibody (A10) was generated from hybridomas. A rabbit anti-Flag monoclonal antibody was purchased from Sigma-Aldrich Corp. (St Louis, MO). Rabbit anti-METTL14, YTHDF2 and ALKBH5 antibodies were purchased from Proteintech Group Inc. METTL14 or EBNA3C knocked down cells were transduced with lentivirus containing specific shRNAs and shRNAs against luciferase were used as a control. The knocked down cell lines were then selected with 1ug/ml puromycin.

### Isolation of m6A RNA fragments

Isolation of m6A-containing fragments was performed as previously described with minor modifications [38]. Briefly, total RNA was extracted from cells using TRIzol Reagent (Invitrogen, Inc., Carlsbad, CA). The total RNA was fragmented in a buffer containing 100 mM Tris-HCl at pH 8.0 and 100 mM ZnCl_2_ followed by incubation at 94°C for 8 min. Successful fragmentation of mRNA with sizes close to 120 nucleotides was validated using a BioRad Geldoc Station (Bio-Rad, Hercules, CA). Before immunoprecipitation, 10 μg of anti-m6A antibody was incubated with 30 μl slurry of Pierce Protein A Agarose beads (Thermo Fisher Scientific, Waltham, MA) in 250 μl PBS with 0.5% BSA at 4°C for 2 h. The beads were washed three times in cold PBS with 0.5% BSA. To isolate the m6A-containing fragments, 900 μg of fragmented total RNA was added to the antibody-bound beads in 250 μl IP buffer supplemented with RNasin Plus RNase inhibitor (Promega Inc, Madison, WI), and the mixture was mixed at 4°C for 2h. The beads were washed four times with 1 ml IP buffer before elution with 100 μl IP buffer supplemented with 6.67 mM of m6A salt (M2780, Sigma-Aldrich, St. Louis, MO). The mixture was incubated for 1 h at 4°C with continuous shaking and the eluate was collected. A second elution was carried out and the eluates were pooled together before purification with 2.5-fold ethanol and 1/10 volume of NaAC pH5.2. RNA was washed with 75% ethanol for twice and dissolved in RNase free H_2_O.

### Preparation of MeRIP-seq complementary DNA library

Purified eluate and Input samples were used for the preparation of libraries and sequencing at the Genome Sequencing Facility of the GATC core at the Washington University St. Louis. Approximately 300 ng of mRNA was used for RNA sequencing (RNA-Seq). The library was prepared using the TruSeq stranded mRNA kit (Illumina, San Diego, CA) according to the manufacturer’s protocol. First, the elute-frag-prime stage was done at 80°C for 2 min to allow annealing without causing fragmentation [12]. RNA was reverse transcribed into first strand cDNA using reverse transcriptase and random primers. This was followed by the second strand cDNA synthesis using DNA Polymerase I and RNase H. The cDNA fragments were used for end repair process with the addition of a single ‘A’ base followed by ligation of adapters. The products were then purified and enriched by PCR amplification for 12 cycles to generate the final RNA-seq library. cDNA libraries were quantified and pooled and subsequent sequencing on an Illumina HiSeq3000 platform 50 bp single read sequencing module.

### Data analysis for MeRIP-seqencing sets

After sequencing, the first step is quality and adapter trimming. Trimming was conducted by Trim Galore (https://www.bioinformatics.babraham.ac.uk/projects/trim_galore/) to remove adapter sequences and low-quality bases (bases with < 20 quality score will be removed). After trimming, the MeRIP-seq reads were aligned to EBV reference genome by the aligner HISAT2 [39] with the annotation of the splice sites (the EBV reference genome and annotation were downloaded from https://www.ncbi.nlm.nih.gov/nuccore/NC_007605.1). The m6A peaks were called by a graphical model-based peak calling method–MeTPeak [40], with the parameters of window width = 50, sliding step = 50 and read length = 50. The peaks were visualized in IGV (http://www.broadinstitute.org/igv).

### RNA immunoprecipitation (RIP)

RIP was performed as previously described [33]. Briefly, 10^7^ LcLs or Akata cells were collected and lysed with RIP buffer (10 mM Tris-HCl pH 7.4, 150 mM NaCl, 5mM EDTA, 0.5mM DTT, 0.5% NP40, 100 U/ml RNAase inhibitor SUPERase•in, 1×protease inhibitor (539131, Millipore, Burlington, MA). 3μg of METTL14 (Proteintech Group Inc, Rosemont, IL) or IgG (NI01, Millipore, Burlington, MA) was conjugated to protein A/G agarose beads (Thermo Fisher Scientific, Waltham, MA) by incubation for overnight at 4°C in RIP buffer at 4°C overnight. Beads were washed with RIP buffer for three times, followed by DNA digestion at 37°C for 30 min and incubation with 50μg of proteinase K (Thermo Fisher Scientific, Waltham, MA) at 55°C for 30min. Input and co-immunoprecipitated RNAs were recovered by TRIzol, extraction and analyzed by qPCR.

### Transfection, Immuno-precipitation and Western blotting

Saos-2 and HEK293 cells were transfected with jetPRIME (Polyplus Transfection, Illkirch, France) according to the manufacturer’s instructions. BJAB cells were transfected by electroporation (220V, 950 μF) with Bio-Rad Gene Pulser II electroporator in 400 ul of serum-free medium. The cells were then transferred to complete RPMI 1640 media, which was preincubated to 37°C.

Immunoprecipitation (IP) and Western blotting were performed as described previously [41]. Briefly, cells were collected and were lysed in lysis buffer (10 mM Tris, 1% NP-40, 2 mM EDTA, 150 mM NaCl [pH 7.5]) with protease inhibitors. For IP, lysates were incubated with the antibodies indicated in the figures and 30 μl of a 1:1 mixture of protein A/G Sepharose beads at 4°C overnight. The beads were washed with RIPA buffer for 3 times, boiled and were subjected to SDS-PAGE for Western blotting.

### Xenograft, Immunohistochemistry, and Immunofluorescence

NOD.CB17-PrkdcSCID (NOD/SCID) male mice were purchased from Jackson Laboratory (Jackson Labs, Bar harbor, ME) at 6 weeks of age and 6 million cells were subcutaneously injected. 3 weeks later, mice were sacrificed and the weight and volume of the tumor were measured.

Immunohistochemistry was performed as described previously [42]. Tissues were embedded in paraffin and sectioned. The slides were treated at 60°C for 60 min and then deparaffinized in xylene and a gradient concentration of ethanol. Antigen was retrieved in boiling Tris buffer (pH 9.0) for 18 min. The slides were immersed in 3% H_2_O_2_ for 10 min, and permeabilized with 0.5% Triton X-100 at RT for 10 min. The slides were blocked in 5% bovine serum albumin (BSA) at RT for 30 min and incubated overnight with diluted primary antibody at 4°C, and then diluted HRP-labeled secondary antibody was added at room temperature for 60 min. After development with diaminobenzidine for 3 min, the slides were washed in water and counterstained with hematoxylin.

For immunofluorescence, cells were seeded on glass coverslips in 24-well plates before transfection. After treatment, cells were fixed with 4% paraformaldehyde at 4°C for 60min and permeated with 0.2% Triton X-100 in PBS for 10min. Nuclei were visualized by staining with DAPI for 2 min. Images were acquired using a Fluoview FV300 confocal microscope and Fluoview software was used for image analysis.

### Dual-luciferase reporter assay

HEK293T cells were co-transfected with pGL4.1 plasmid with METTL14 promoter, pRL-TK (Promega, Madison, WI, USA), Myc-tagged EBNA3C, or empty plasmids. Cells were harvested and the dual-luciferase reporter assay was performed according to the manufacturer's instructions (Promega, Madison, WI, USA) 48 hours after transfection. Luciferase value was read using a Cytation 5 (BioTek, Winooski, VT, USA).

### RNA isolation and Real-time PCR

The total RNA extraction was performed using Trizol reagent (Invitrogen, Inc., Carlsbad, CA) and treated with DNase I (Invitrogen, Inc., Carlsbad, CA). cDNA was prepared with Superscript II reverse transcriptase kit (Invitrogen, Inc., Carlsbad, CA) according to the manufacturer’s protocol. Quantitative real-time PCR analysis was performed by using SYBR green real-time master mix (MJ Research Inc., Waltham, MA). The primers used are listed in S5 Table.

### CSFE proliferation assay

Cells were collected and suspended in 1XPBS at a concentration of 1 million cells/ml. The CFSE solution was added to make a final concentration of 5μM. An equal volume of 1XPBS containing 5% FBS was added after 10 min incubation at room temperature. Cells were washed three times with 1XPBS containing 5% FBS and equally divided into several plates for incubation. 3 days later, cells were harvested, washed with ice-cold 1XPBS and resuspended in 5ml 1XPBS, then run on FACS Calibur cytometer (Becton-Dickinson Inc., San Jose, CA) followed by analysis with FlowJo software (Treestar, Inc., San Carlos, CA).

### Colony formation and Soft agar assays

HEK293 or Saos-2 cells were transfected with control vector, Myc-EBNA3C or Flag-METTL14 and allowed to grow in DMEM supplemented with 1mg/ml G418 (Sigma-Aldrich, St. Louis, MO, USA). After two weeks of selection, 10^5^ cells were seeded in 6-well plates and allowed to grow for 5 days. Cells were stained with 0.005% Crystal Violet overnight and scanned by PhosphorImager (Molecular Dynamics, Piscataway, NJ) and the area of the colonies measured by using Image J software (Adobe Inc., San Jose, CA). Three independent experiments were performed. The soft agar assays were performed using LcLs and Burkitt lymphoma cells. Briefly, 1 ml of 0.5% agar in RPMI media was poured into 6-well plates and set aside to solidify. 0.5 ml 0.3% agar/medium containing 2×10^5^ cells were added to the plates as the middle layer. Then cells were covered with a top layer of another 1ml 0.5% agar/medium. After two weeks, colonies were stained with 0.005% crystal violet for 1 hour and scanned using a Licor Odyssey system (LiCor Inc., Lincoln, NE). The number of colonies was counted using ImageJ software (Adobe Inc., San Jose, CA).

### Statistical analysis

Each experiment was repeated at least three times. The mean scores were examined by using Student’s *t-*test. All statistical tests were performed using Microsoft Office Excel. A *P-*value of 0.05 was considered to be a statistically significant difference. A *P-*value of 0.01 was considered to indicate highly statistical significance.

## Supporting information

S1 Fig Relative amounts of latent and lytic transcripts in input mRNA. **(A-C)** The transcription level of indicated genes in the input sample of LcLs. Viral gene transcription levels were detected and normalized to cellular control GAPDH. The relative amount of EBNA1 expression was set as 1. **(D)** RPKM: Reads Per Kilobase per Million mapped reads of indicated genes from RNA seq data of LcLs. DNA contamination: Detection of DNA in input RNA samples before reverse transcription.
(TIF)

S2 Fig Measurement of METTL14 with and without actinomycin D treatment in uninduced LcLs. **(A)** DMSO or **(B)** Actinomycin D was used to inhibit transcription for 6 and 12 hours and the levels of METTL14 mRNA was determined. Experiments were independently repeated three times, and results are presented as mean±s.d. from the three experiments.
(TIF)

S3 Fig The transcription levels of m6A related genes in EBV negative and positive cells. 3 million B-cell, LcLs **(A)**, Akata EBV negative cell and Akata EBV-positive cells **(B)** were collected and total RNA extraction was performed using Trizol reagent and treated with DNase I, then cDNA was prepared with Superscript II reverse transcriptase kit. Genes transcription level was detected and normalized to a cellular control GAPDH RNA. ΔΔCt method was used to analyze qPCR data. Error bars represent standard deviation. Experiments were independently repeated three times, and results are presented as mean±s.d. from the three experiments. “**” represents p-value <0.01; “*” represents p-value <0.05.
(TIF)

S4 Fig The quantitation of METTL14 and EBNA3C protein levels shown in Fig 3. Fold change means relative densities which were quantified using the Odyssey ImageQuant software. This was representative of experiments repeated for each panel with similar results. UI: uninduced; IN: induced. **(A-F)** The quantitation of METTL14 protein levels shown in Fig 3A–3F respectively. **(G-L)** The quantitation of EBNA3C protein levels shown in Fig 3G–3L respectively.
(TIF)

S5 Fig The functions of METTL14, METTL3 and demethylase inhibitor activities on infection with EBV. **(A)** LcLs with shRNA cr or shMETTL14 were treated with DMSO or TPA (20 ng/ml) and Butyric acid (BA, 2.5 mM) for indicated time. Cells were harvested at various times (0, 24, 48, 72, 96 and 120h) and EBNA1 primers were used for determination of viral copy number. **(B)** RIP using METTL3 antibody to detect the overall levels of METTL3 on viral genes in LcLs. Primers were designed for the indicated gene regions. **(C)** 5 million LcL shCr, LcL shMETTL3 (shM3) cells were collected, lysed and subjected to western blot with indicated antibodies. **(D)** The effects of the demethylase inhibitor on EBV latent and lytic gene expression. 5 million LcLs were treated with TPA and Butyric acid (IN) or DMSO (UI), with or without meclofenamic acid, for 48 hours. Cells were collected, lysed and subjected to western blot with indicated antibodies. UI: uninduced with drugs; IN: induced with drugs.
(TIF)

S6 Fig EBNA3C regulates METTL14 expression at the transcription level. **(A-G)** 5 million Saos-2 cells were transfected with control plasmids, Myc tagged EBNA2 (E2), LMP1 (L1), LMP2A (L2A), LMP2B (L2B), EBNA3A (E3A), EBNA3B (E3B) or EBNA3C (E3C). 48 hours later, cells were collected, lysed and subjected to western blot with indicated antibodies and METTL14 levels were quantitated. GAPDH (GAP) was used as the loading control. (**H-I)** 5 million BJAB, BJAB7 (B7), BJAB10 (B10), B cell, LcL shCr, and LcL shEBNA3C cells were collected, lysed and subjected to western blot with indicated antibodies and METTL14 levels were quantitated. **(J-K)** 5 million BJAB, BJAB7 (B7), BJAB10 (B10), B cell, LcL shCr, and LcL shEBNA3C cells were collected and total RNA was extracted with Trizol reagent. The cDNA was prepared with reverse transcriptase kit, and EBNA3C and METTL14 mRNA was detected by RT-qPCR. GAPDH (GAP) was set as an internal reference. **(L-M)** HEK293 and Saos-2 cells were transfected with the reporter constructs containing the wild-type METTL14 promoter and an increasing amount of Myc-EBNA3C. Cells were collected and lysed in lysis buffer at 48 hours post-transfection. Luciferase activity was measured according to the dual-luciferase reporter assay kit compared to pGL4 vector control. The cell lysate was resolved by 10% SDS-PAGE to monitor EBNA3C expression. GAPDH western blot was used as an internal loading control. Experiments were independently repeated three times, and results are presented as mean±s.d. from the three experiments. “**” represents p-value <0.01; “*” represents p-value <0.05.
(TIF)

S7 Fig A control for METTL14-IP in Fig 4H. HEK293 cells were transfected with an empty vector carrying a Flag tag. Flag antibody was used to do immunoprecipitations to exclude any non-specific binding in the control group. For blotting, Myc antibody was used to monitor the expression of EBNA3C and the possible pulled down EBNA3C truncates. Flag antibody was used to monitor the expression of Flag-associated proteins. METTL14 antibody was used to monitor the expression of METTL14 in different samples.
(TIF)

S8 Fig The truncated regions of EBNA3C have no effect on the expression, protein stability and oncogene function of METTL14. **(A)** 30 million HEK293 cells were transfected with indicated plasmids and lysed with RIPA buffer 48 hours later, and samples were resolved by 10% SDS-PAGE and proteins were detected by the specific antibodies. **(B-C)** 10 million HEK293 cells were co-transfected with control plasmids, EBNA3C100-200 (B) and EBNA3CΔ100–200 (C), and Flag-METTL14 expression plasmids. 36 hours later, cells were treated with 40 μg/ml cycloheximide (CHX) for 0, 4, 8, 12 hours, and then cells were collected, lysed and subjected to western blot with indicated antibodies. **(D-G)** BJAB cells were transfected with control vector, Myc-EBNA3C, Myc-EBNA3C mutants, Flag-METTL14 or Myc-EBNA3C plus Flag-METTL14 and allowed to grow in DMEM supplemented with 1mg/ml G418. The same number of G418 selected cells were seeded to 6-well plates, collected and lysed in lysis buffer after 5 days culture. The lysates were subjected to western blots with indicated antibodies (D). 1X10^5^ cells were stained with 5μM CFSE for 10 minutes at 37°C. The cells were washed, cultured, and harvested after culturing for 3 days. Flow cytometry was used to analyze CFSE-labeled cells (F). The transfected cells were assessed for their ability to promote colony formation using the soft agar assays (E and G).
(TIF)

S9 Fig EBNA3C interacts and colocalizes with METTL14 in human cells. **(A-D)** HEK293 or Saos-2 cells were seeded on coverslips and transfected with Myc-EBNA3C using jetPRIME transfection reagent. The cells were fixed with 4% PFA, stained with specific antibodies. EBNA3C was detected by mouse anti-Myc (9E10) antibodies followed by the secondary anti-mouse Alexa Fluor 594. METTL14 were detected by rabbit anti-METTL14 antibodies, followed by anti-rabbit Alexa Fluor 488 as the secondary antibody. The nuclei were subsequently stained with DAPI, and the images were captured using an Olympus Fluoview confocal microscope.
(TIF)

S10 Fig Oncogenic assay for expression of METTL14, EBNA3C in BJAB cells. BJAB cells were transfected with control vector, Myc-EBNA3C, Flag-METTL14 or Myc-EBNA3C plus Flag-METTL14 and allowed to grow in DMEM supplemented with 1mg/ml G418. The same number of G418 selected cells were seeded to 6-well plates, collected and lysed in lysis buffer after 5 days in culture. The lysates were subjected to western blot with indicated antibodies **(A)**. 1X105 cells were stained with 5μM CFSE for 10 minutes at 37°C. The cells were washed, cultured, and harvested after culturing for 3 days. Flow cytometry was used to analyze CFSE-labeled cells **(C)**. The transfected cells were assessed for their ability to promote colony formation using the soft agar assays **(B and D)**.
(TIF)

S11 Fig Knock-down of METTL14 or EBNA3C led to decreased tumor growth. 6 million LcL shCr, LcL shMETTL14, LcL shEBNA3C, and LcL shMETTL14 plus shEBNA3C were subcutaneously injected into NOD-SCID mice to assess the effect of knocking down METTL14 or EBNA3C on tumor growth *in vivo*. 3 weeks later, mice were sacrificed and pictures were taken **(A)**.The volume of the tumors was measured every 2 days **(B)**.
(TIF)

S12 Fig Mutations in m6A sites of EBNA3C demonstrates a loss of function of EBNA3C in cell growth. **(A)** Mutations within the consensus site of four identified m6A sites in the EBNA3C gene were introduced into construct E3C_mut_. The framed base pairs were considered as containing the DRACH motif. **(B)** The wild-type EBNA3C and mutant EBNA3C were transfected into BJAB cells and Raji cells. 48 hours later, the expression of wild-type EBNA3C and mutant EBNA3C were detected with western blot. **(C-D)** Wild-type EBNA3C or mutant EBNA3C were assessed for their ability to promote colony formation using the soft agar assays. Photomicrograph of representative colonies from three independent experiments is shown. **(E)** Wild-type EBNA3C or mutant EBNA3C were assessed for their ability to promote cell growth with CFSE staining assay. Experiments were independently repeated three times, and results are presented as mean±s.d. from the three experiments. “**” represents p-value <0.01; “*” represents p-value <0.05.
(TIF)

S1 Table M6A peak information in LcLs. (XLS)

S2 Table M6A peak information in Akata cells. (XLS)

S3 Table M6A peak information in lytic LcLs. (XLS)

S4 Table M6A peak information in lytic Akata cells. (XLS)

S5 Table Primers used in the experiments. (XLSX)
