# Supplementary figures and images for: EBV epitranscriptome reprogramming by METTL14 is critical for viral-associated tumorigenesis
Source: PLoS Pathog. 2019 Jun 21;15(6):e1007796. doi: 10.1371/journal.ppat.1007796 (PMC6588254; doi:10.1371/journal.ppat.1007796)

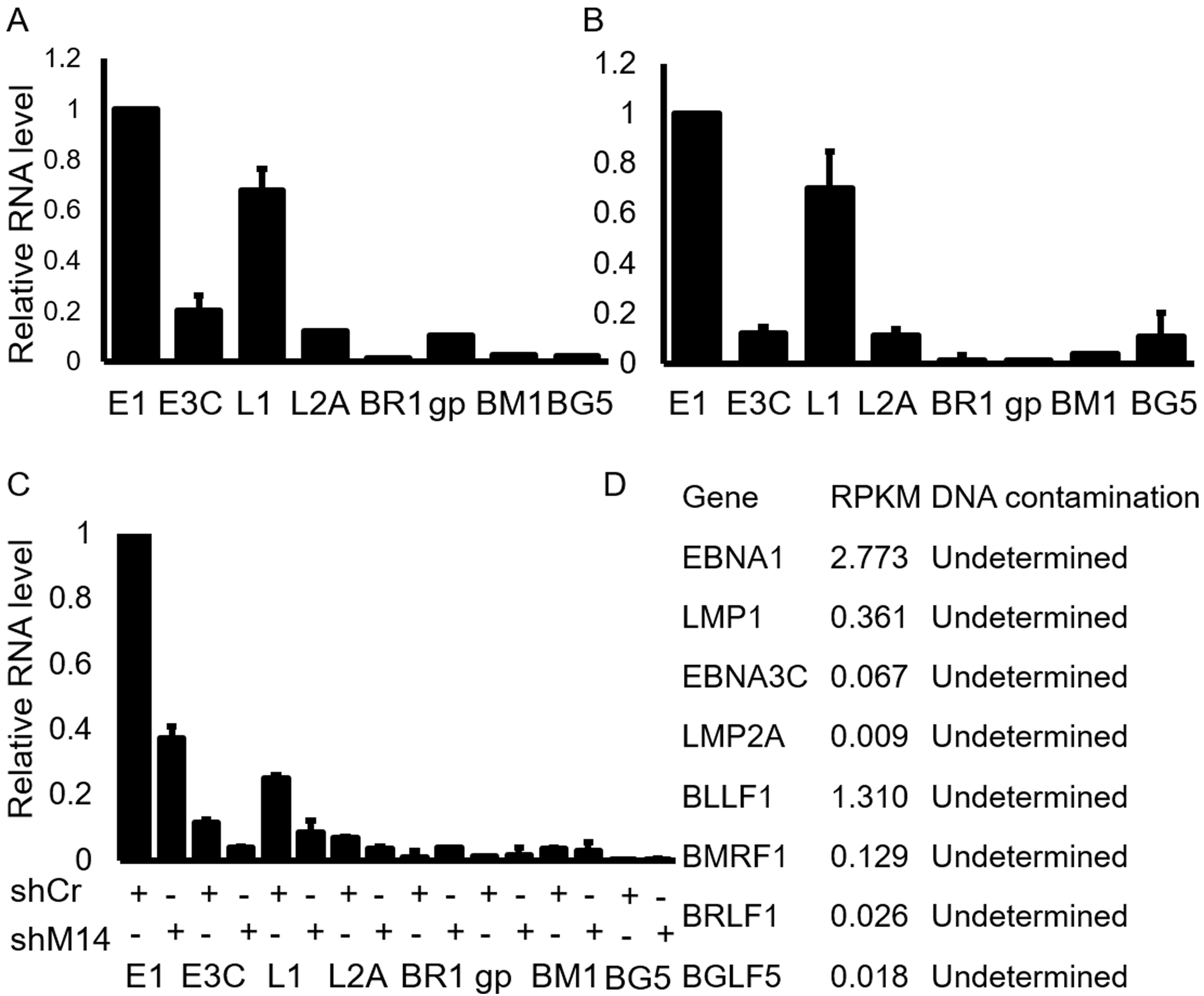

Supplement: S1 Fig — (A-C) The transcription level of indicated genes in the input sample of LcLs. Viral gene transcription levels were detected and normalized to cellular control GAPDH. The relative amount of EBNA1 expression was set as 1. (D) RPKM: Reads Per Kilobase per Million mapped reads of indicated genes from RNA seq data of LcLs. DNA contamination: Detection of DNA in input RNA samples before reverse transcription. (TIF) [file ppat.1007796.s001.tif]

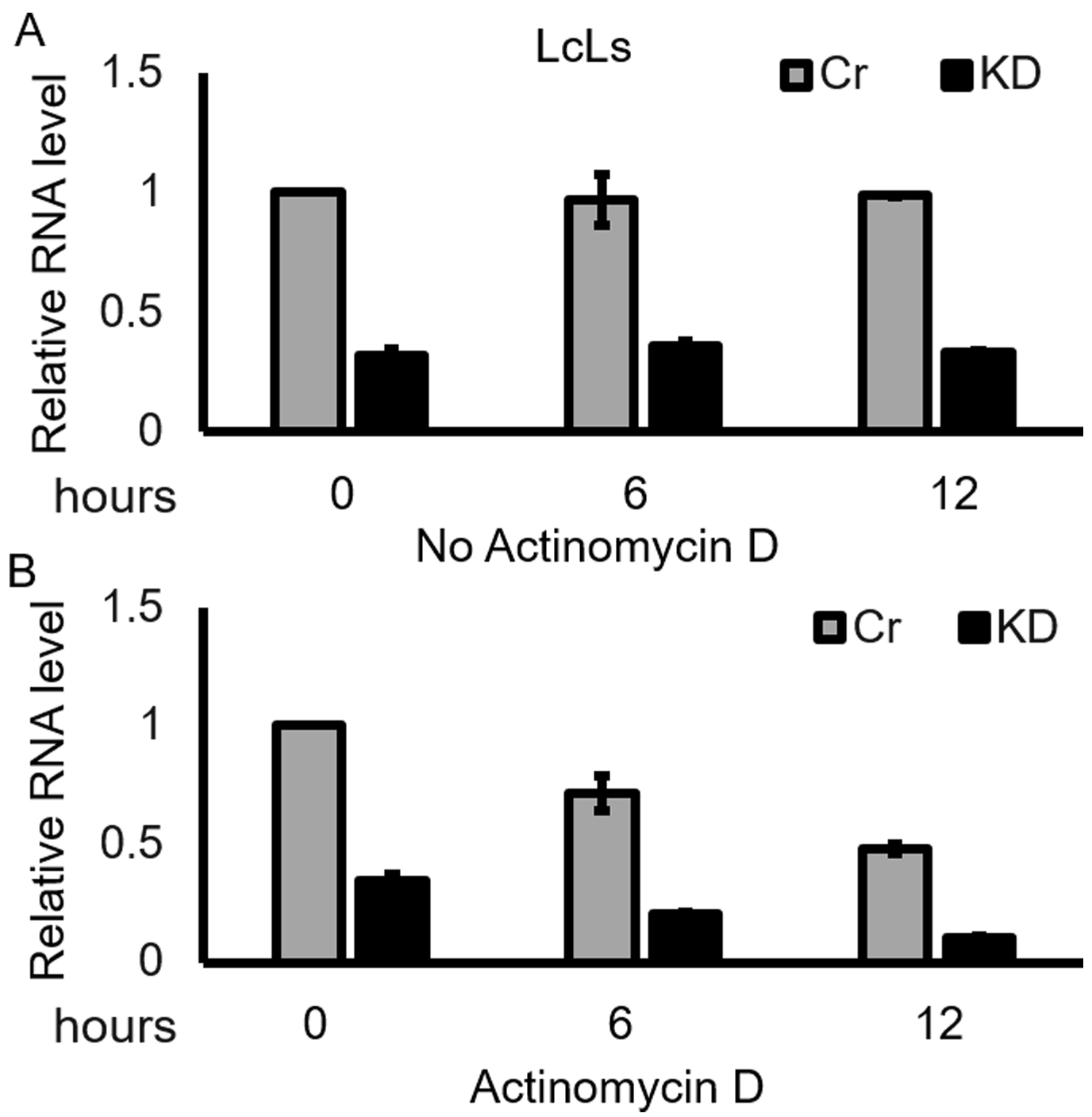

Supplement: S2 Fig — (A) DMSO or (B) Actinomycin D was used to inhibit transcription for 6 and 12 hours and the levels of METTL14 mRNA was determined. Experiments were independently repeated three times, and results are presented as mean±s.d. from the three experiments. (TIF) [file ppat.1007796.s002.tif]

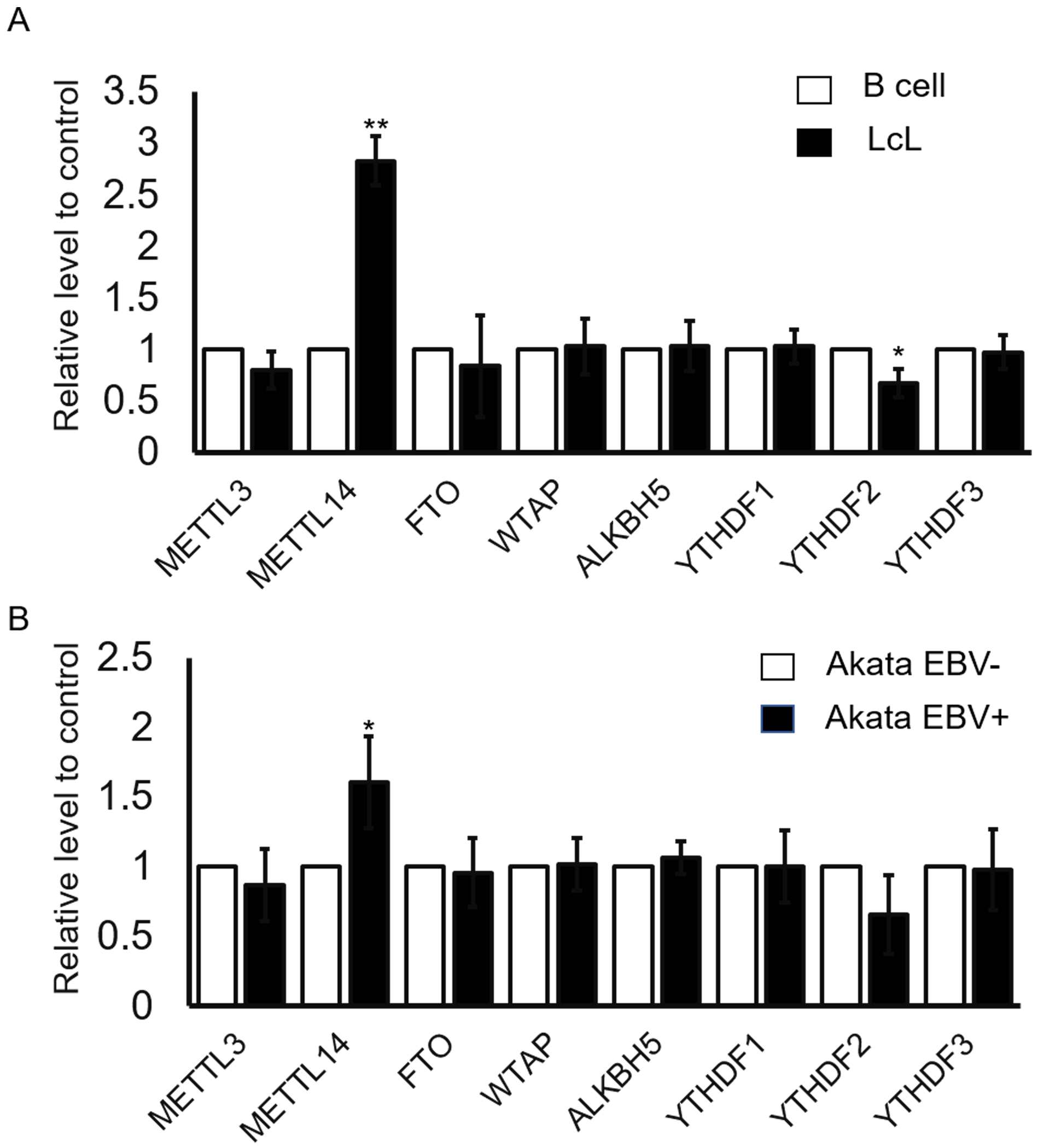

Supplement: S3 Fig — 3 million B-cell, LcLs (A), Akata EBV negative cell and Akata EBV-positive cells (B) were collected and total RNA extraction was performed using Trizol reagent and treated with DNase I, then cDNA was prepared with Superscript II reverse transcriptase kit. Genes transcription level was detected and normalized to a cellular control GAPDH RNA. ΔΔCt method was used to analyze qPCR data. Error bars represent standard deviation. Experiments were independently repeated three times, and results are presented as mean±s.d. from the three experiments. “**” represents p-value <0.01; “*” represents p-value <0.05. (TIF) [file ppat.1007796.s003.tif]

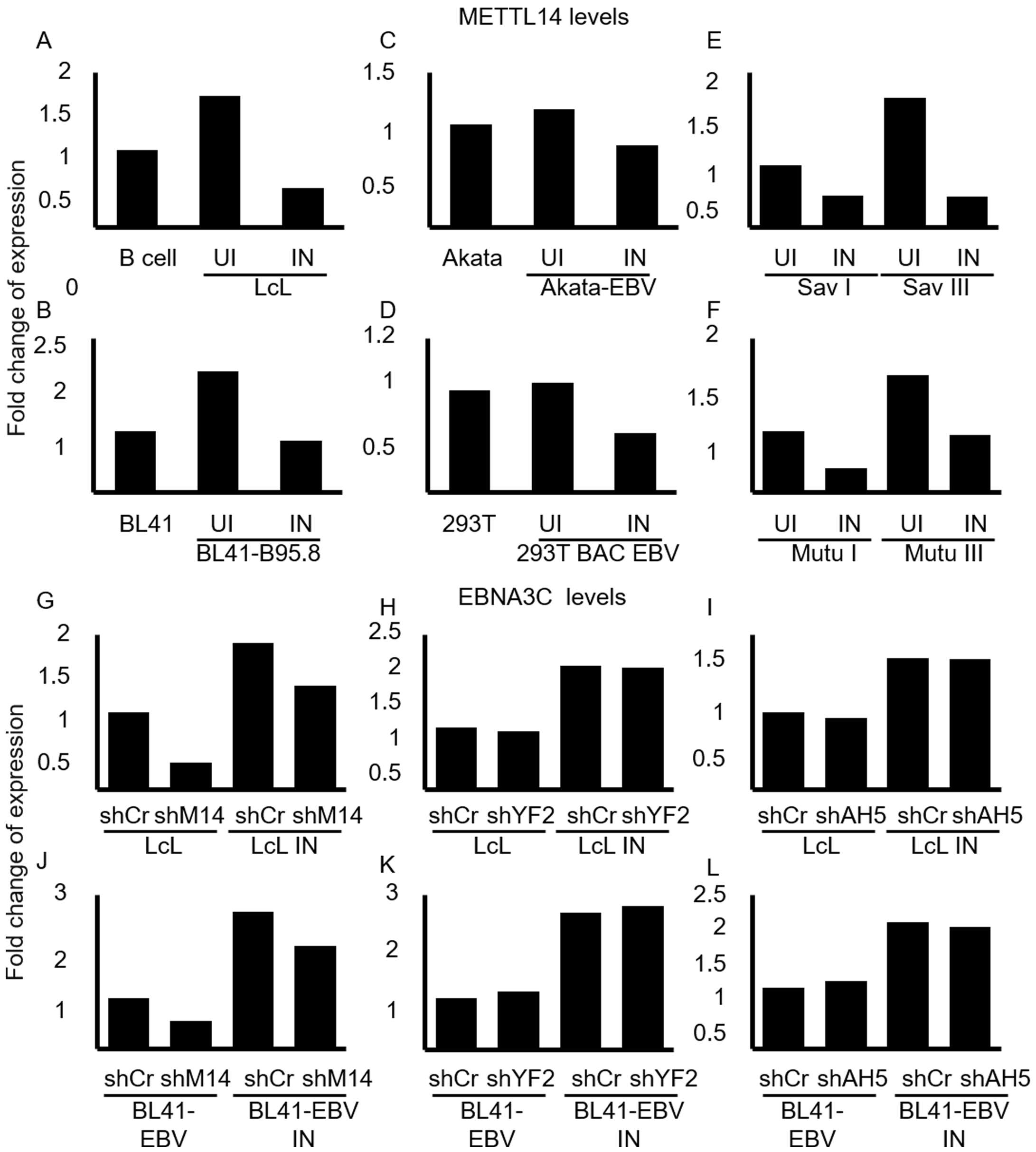

Supplement: S4 Fig — Fold change means relative densities which were quantified using the Odyssey ImageQuant software. This was representative of experiments repeated for each panel with similar results. UI: uninduced; IN: induced. (A-F) The quantitation of METTL14 protein levels shown in Fig 3A–3F respectively. (G-L) The quantitation of EBNA3C protein levels shown in Fig 3G–3L respectively. (TIF) [file ppat.1007796.s004.tif]

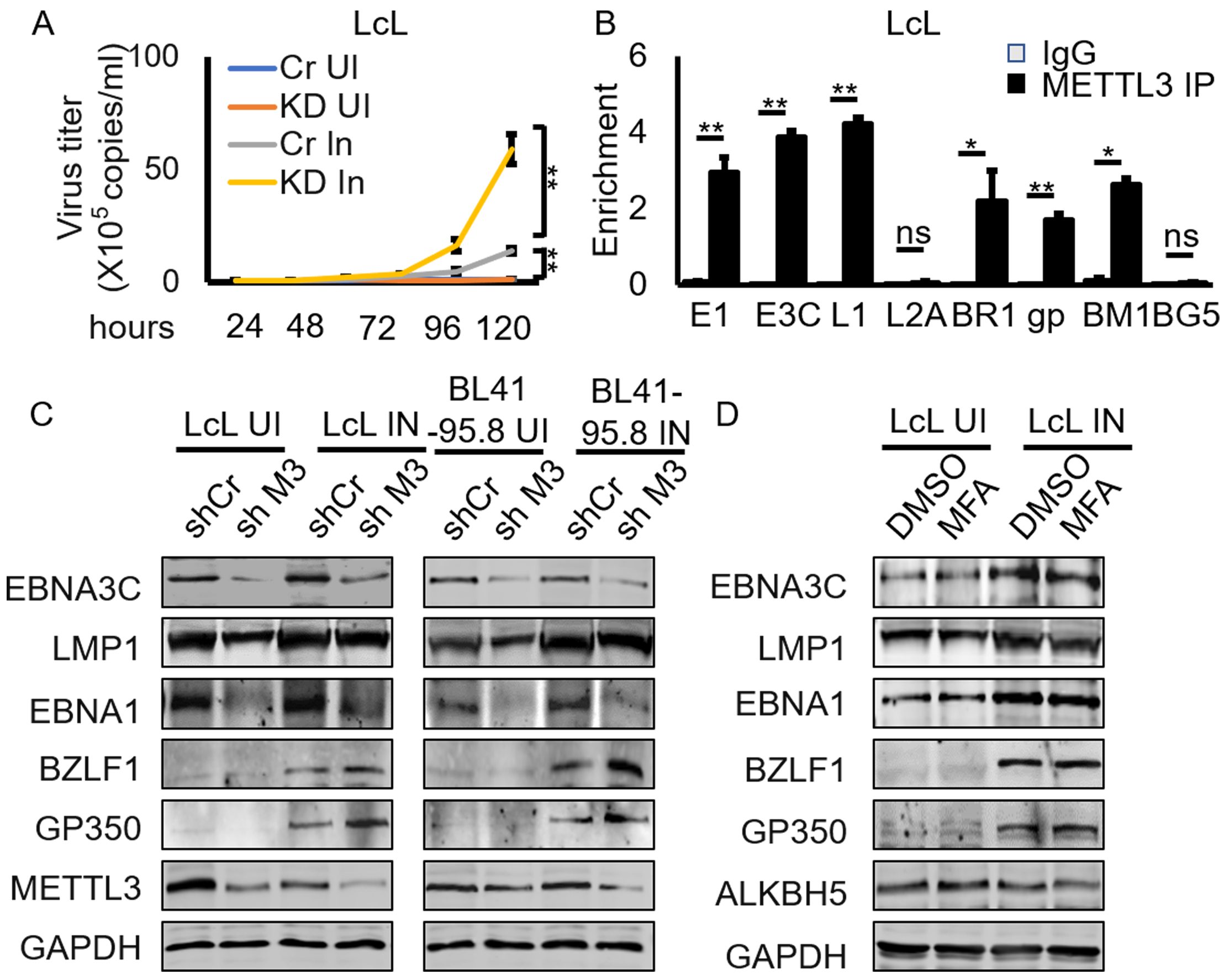

Supplement: S5 Fig — (A) LcLs with shRNA cr or shMETTL14 were treated with DMSO or TPA (20 ng/ml) and Butyric acid (BA, 2.5 mM) for indicated time. Cells were harvested at various times (0, 24, 48, 72, 96 and 120h) and EBNA1 primers were used for determination of viral copy number. (B) RIP using METTL3 antibody to detect the overall levels of METTL3 on viral genes in LcLs. Primers were designed for the indicated gene regions. (C) 5 million LcL shCr, LcL shMETTL3 (shM3) cells were collected, lysed and subjected to western blot with indicated antibodies. (D) The effects of the demethylase inhibitor on EBV latent and lytic gene expression. 5 million LcLs were treated with TPA and Butyric acid (IN) or DMSO (UI), with or without meclofenamic acid, for 48 hours. Cells were collected, lysed and subjected to western blot with indicated antibodies. UI: uninduced with drugs; IN: induced with drugs. (TIF) [file ppat.1007796.s005.tif]

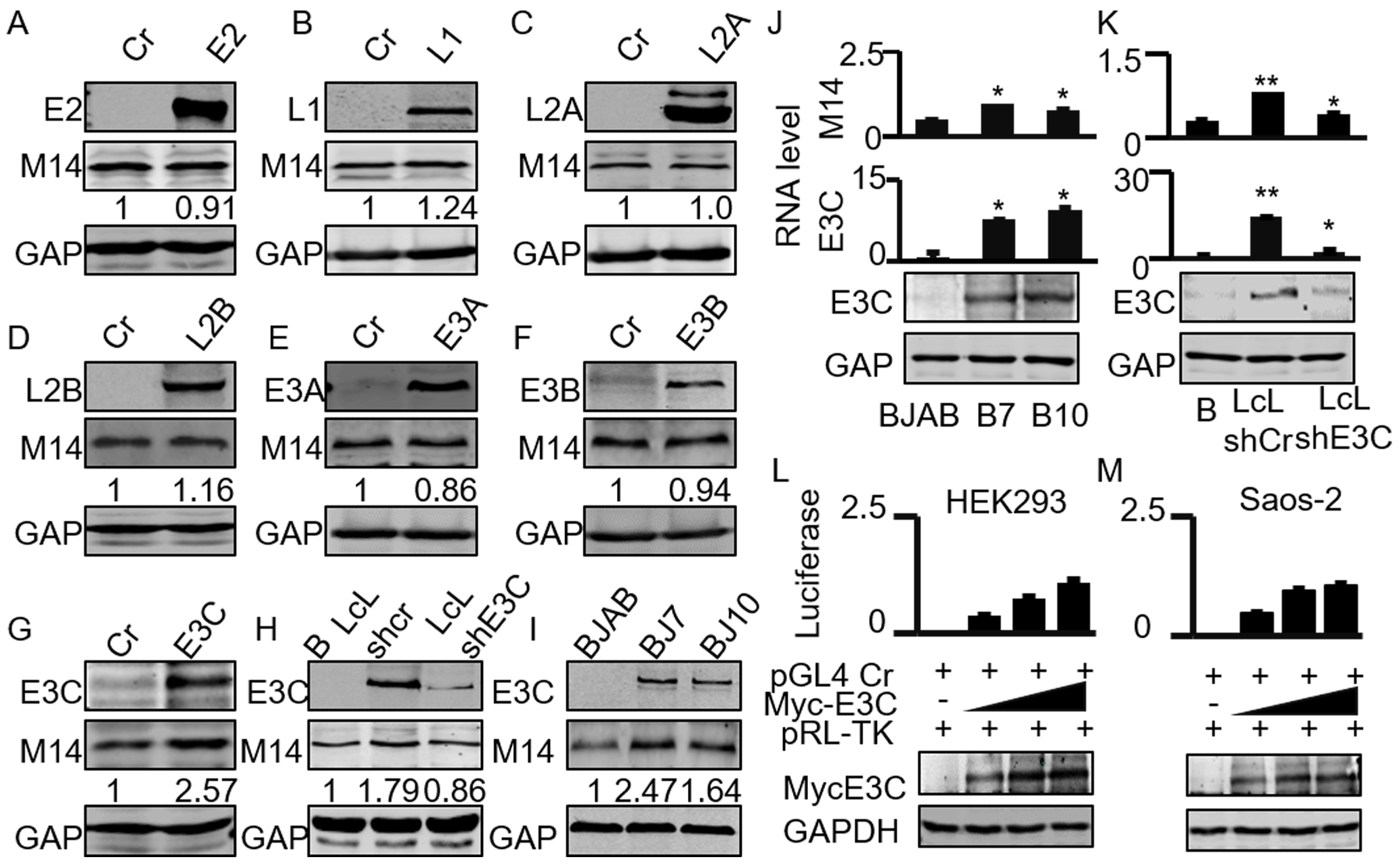

Supplement: S6 Fig — (A-G) 5 million Saos-2 cells were transfected with control plasmids, Myc tagged EBNA2 (E2), LMP1 (L1), LMP2A (L2A), LMP2B (L2B), EBNA3A (E3A), EBNA3B (E3B) or EBNA3C (E3C). 48 hours later, cells were collected, lysed and subjected to western blot with indicated antibodies and METTL14 levels were quantitated. GAPDH (GAP) was used as the loading control. (H-I) 5 million BJAB, BJAB7 (B7), BJAB10 (B10), B cell, LcL shCr, and LcL shEBNA3C cells were collected, lysed and subjected to western blot with indicated antibodies and METTL14 levels were quantitated. (J-K) 5 million BJAB, BJAB7 (B7), BJAB10 (B10), B cell, LcL shCr, and LcL shEBNA3C cells were collected and total RNA was extracted with Trizol reagent. The cDNA was prepared with reverse transcriptase kit, and EBNA3C and METTL14 mRNA was detected by RT-qPCR. GAPDH (GAP) was set as an internal reference. (L-M) HEK293 and Saos-2 cells were transfected with the reporter constructs containing the wild-type METTL14 promoter and an increasing amount of Myc-EBNA3C. Cells were collected and lysed in lysis buffer at 48 hours post-transfection. Luciferase activity was measured according to the dual-luciferase reporter assay kit compared to pGL4 vector control. The cell lysate was resolved by 10% SDS-PAGE to monitor EBNA3C expression. GAPDH western blot was used as an internal loading control. Experiments were independently repeated three times, and results are presented as mean±s.d. from the three experiments. “**” represents p-value <0.01; “*” represents p-value <0.05. (TIF) [file ppat.1007796.s006.tif]

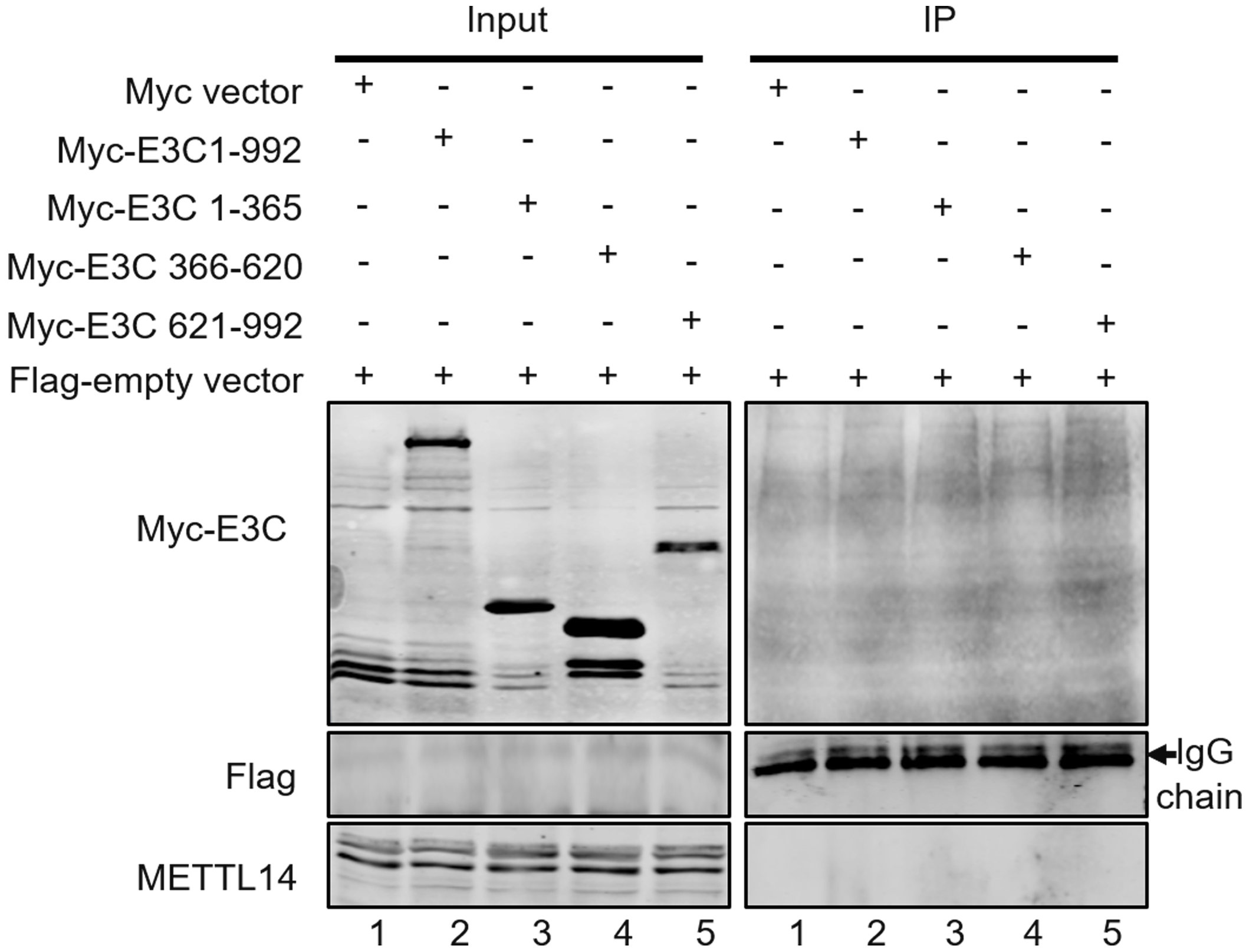

Supplement: S7 Fig — HEK293 cells were transfected with an empty vector carrying a Flag tag. Flag antibody was used to do immunoprecipitations to exclude any non-specific binding in the control group. For blotting, Myc antibody was used to monitor the expression of EBNA3C and the possible pulled down EBNA3C truncates. Flag antibody was used to monitor the expression of Flag-associated proteins. METTL14 antibody was used to monitor the expression of METTL14 in different samples. (TIF) [file ppat.1007796.s007.tif]

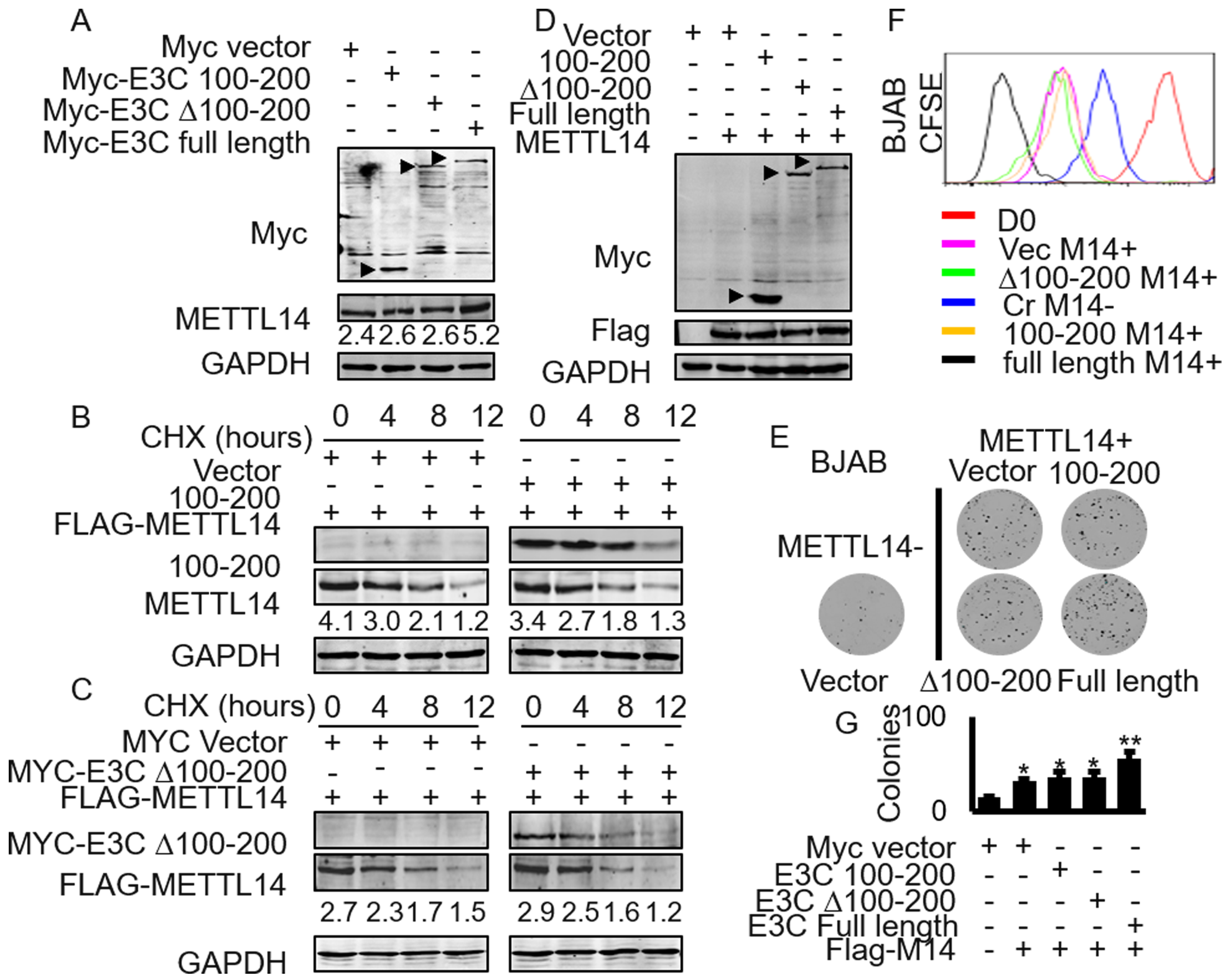

Supplement: S8 Fig — (A) 30 million HEK293 cells were transfected with indicated plasmids and lysed with RIPA buffer 48 hours later, and samples were resolved by 10% SDS-PAGE and proteins were detected by the specific antibodies. (B-C) 10 million HEK293 cells were co-transfected with control plasmids, EBNA3C100-200 (B) and EBNA3CΔ100–200 (C), and Flag-METTL14 expression plasmids. 36 hours later, cells were treated with 40 μg/ml cycloheximide (CHX) for 0, 4, 8, 12 hours, and then cells were collected, lysed and subjected to western blot with indicated antibodies. (D-G) BJAB cells were transfected with control vector, Myc-EBNA3C, Myc-EBNA3C mutants, Flag-METTL14 or Myc-EBNA3C plus Flag-METTL14 and allowed to grow in DMEM supplemented with 1mg/ml G418. The same number of G418 selected cells were seeded to 6-well plates, collected and lysed in lysis buffer after 5 days culture. The lysates were subjected to western blots with indicated antibodies (D). 1X105 cells were stained with 5μM CFSE for 10 minutes at 37°C. The cells were washed, cultured, and harvested after culturing for 3 days. Flow cytometry was used to analyze CFSE-labeled cells (F). The transfected cells were assessed for their ability to promote colony formation using the soft agar assays (E and G). (TIF) [file ppat.1007796.s008.tif]

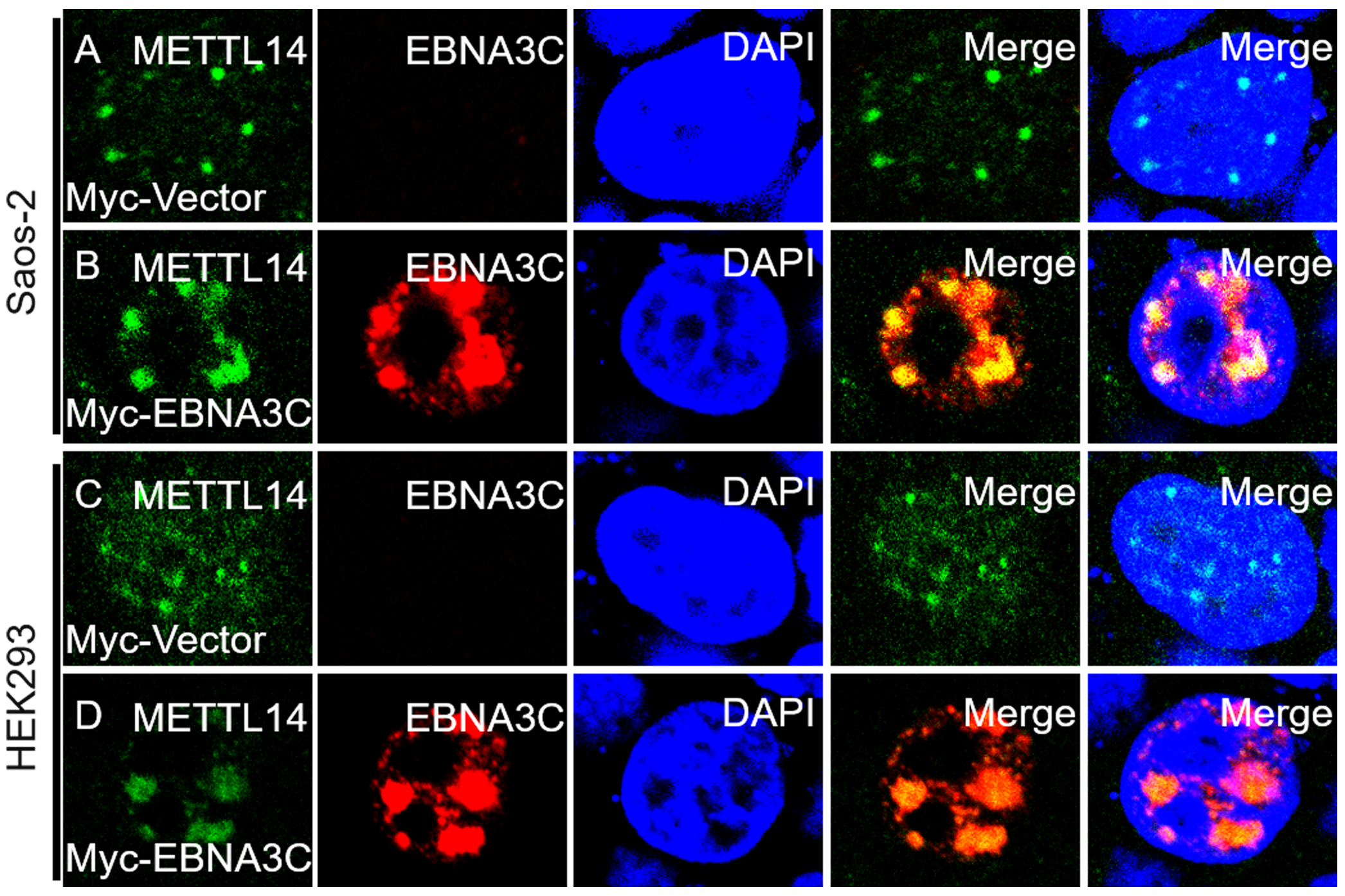

Supplement: S9 Fig — (A-D) HEK293 or Saos-2 cells were seeded on coverslips and transfected with Myc-EBNA3C using jetPRIME transfection reagent. The cells were fixed with 4% PFA, stained with specific antibodies. EBNA3C was detected by mouse anti-Myc (9E10) antibodies followed by the secondary anti-mouse Alexa Fluor 594. METTL14 were detected by rabbit anti-METTL14 antibodies, followed by anti-rabbit Alexa Fluor 488 as the secondary antibody. The nuclei were subsequently stained with DAPI, and the images were captured using an Olympus Fluoview confocal microscope. (TIF) [file ppat.1007796.s009.tif]

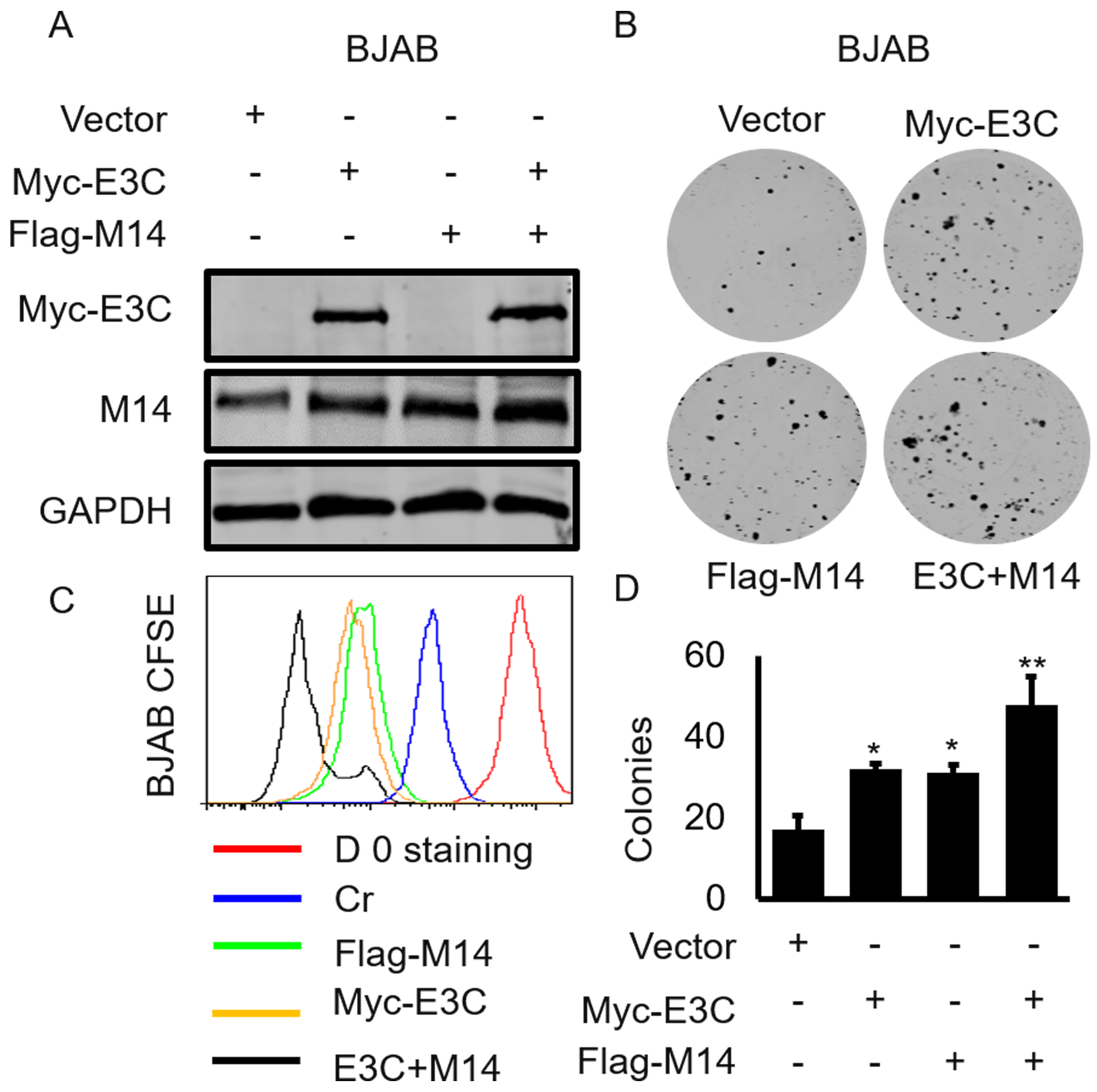

Supplement: S10 Fig — BJAB cells were transfected with control vector, Myc-EBNA3C, Flag-METTL14 or Myc-EBNA3C plus Flag-METTL14 and allowed to grow in DMEM supplemented with 1mg/ml G418. The same number of G418 selected cells were seeded to 6-well plates, collected and lysed in lysis buffer after 5 days in culture. The lysates were subjected to western blot with indicated antibodies (A). 1X105 cells were stained with 5μM CFSE for 10 minutes at 37°C. The cells were washed, cultured, and harvested after culturing for 3 days. Flow cytometry was used to analyze CFSE-labeled cells (C). The transfected cells were assessed for their ability to promote colony formation using the soft agar assays (B and D). (TIF) [file ppat.1007796.s010.tif]

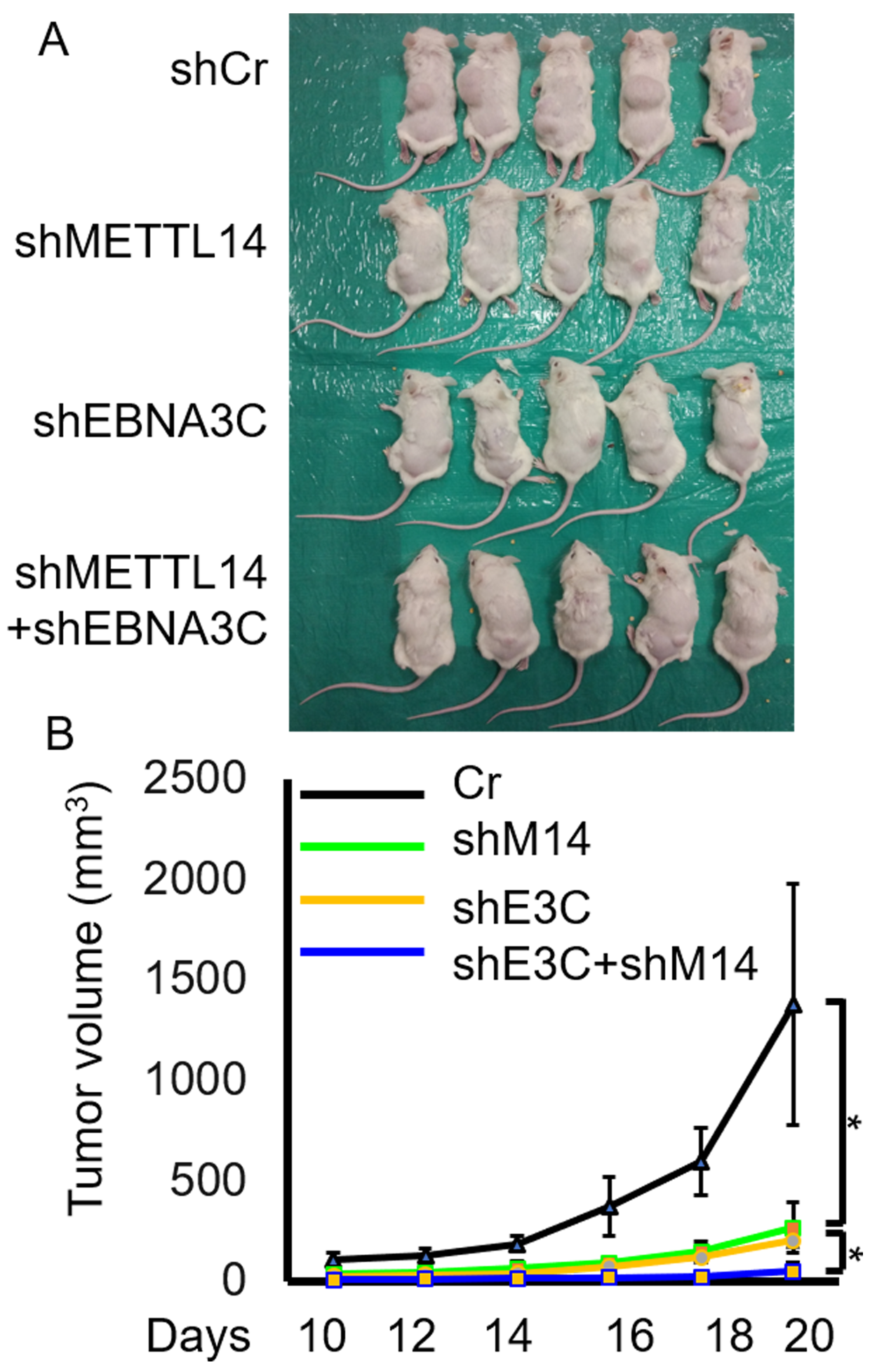

Supplement: S11 Fig — 6 million LcL shCr, LcL shMETTL14, LcL shEBNA3C, and LcL shMETTL14 plus shEBNA3C were subcutaneously injected into NOD-SCID mice to assess the effect of knocking down METTL14 or EBNA3C on tumor growth in vivo. 3 weeks later, mice were sacrificed and pictures were taken (A).The volume of the tumors was measured every 2 days (B). (TIF) [file ppat.1007796.s011.tif]

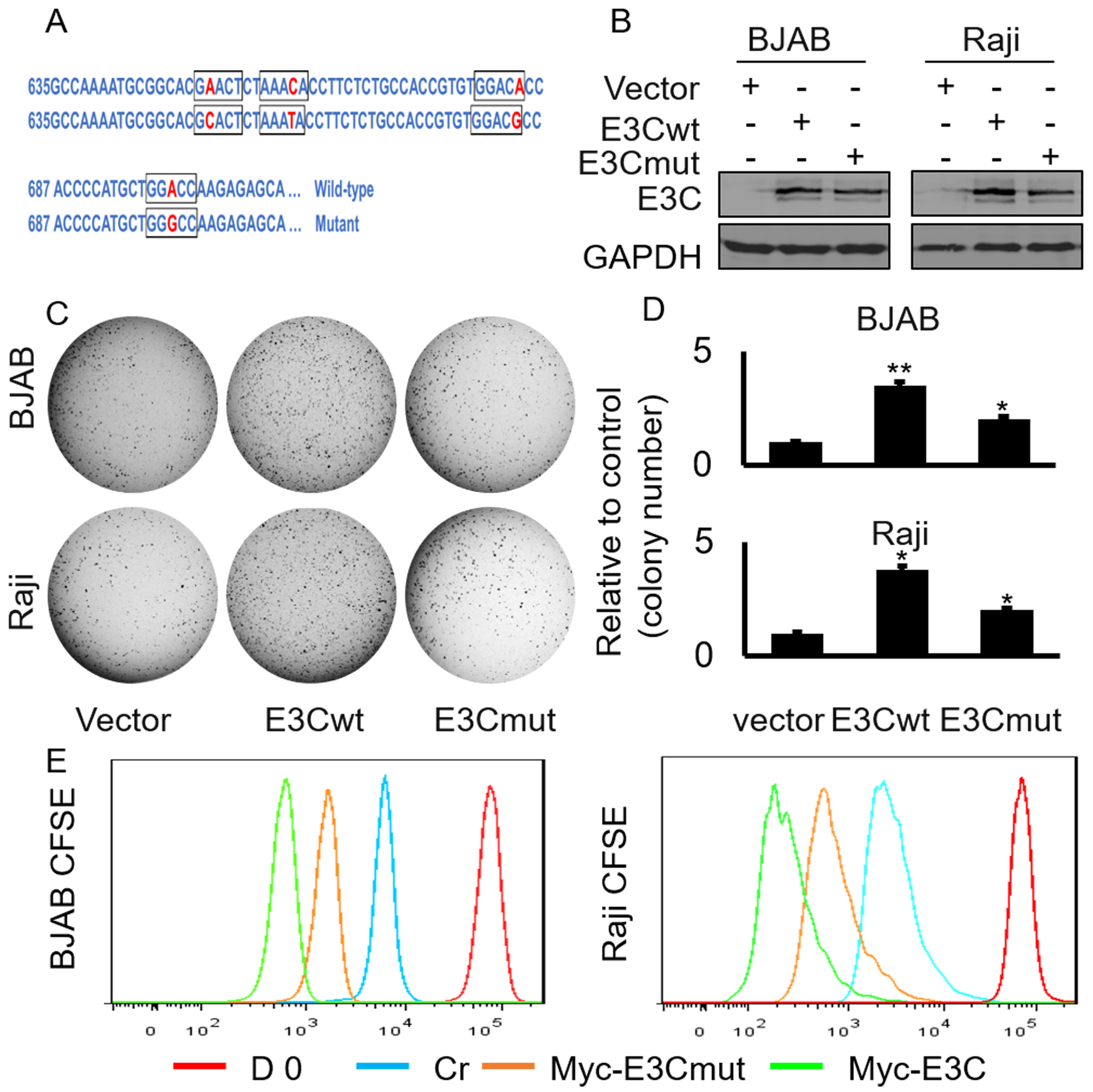

Supplement: S12 Fig — (A) Mutations within the consensus site of four identified m6A sites in the EBNA3C gene were introduced into construct E3Cmut. The framed base pairs were considered as containing the DRACH motif. (B) The wild-type EBNA3C and mutant EBNA3C were transfected into BJAB cells and Raji cells. 48 hours later, the expression of wild-type EBNA3C and mutant EBNA3C were detected with western blot. (C-D) Wild-type EBNA3C or mutant EBNA3C were assessed for their ability to promote colony formation using the soft agar assays. Photomicrograph of representative colonies from three independent experiments is shown. (E) Wild-type EBNA3C or mutant EBNA3C were assessed for their ability to promote cell growth with CFSE staining assay. Experiments were independently repeated three times, and results are presented as mean±s.d. from the three experiments. “**” represents p-value <0.01; “*” represents p-value <0.05. (TIF) [file ppat.1007796.s012.tif]
